# Estrogen receptor β deficiency impairs gut microbiota: a possible mechanism of IBD-induced anxiety-like behavior

**DOI:** 10.1186/s40168-022-01356-2

**Published:** 2022-09-29

**Authors:** Yuanyuan Ma, Tianyao Liu, Xin Li, Anqi Kong, Rui Xiao, Ruxin Xie, Junwei Gao, Zhongke Wang, Yun Cai, Jiao Zou, Ling Yang, Lian Wang, Jinghui Zhao, Haiwei Xu, Warner Margaret, Xingshun Xu, Jan-Ake Gustafsson, Xiaotang Fan

**Affiliations:** 1 Department of Military Cognitive Psychology, School of Psychology, Third Military Medical University, Chongqing, China; 2 Institute of Neuroscience, Soochow University, Suzhou, China; 3 Southwest Eye Hospital, Southwest Hospital, Third Military Medical University, Chongqing, China; 4 Center for Innovative Medicine, Department of Biosciences and Nutrition, Karolinska Institute, Stockholm, Sweden; 5 Center for Nuclear Receptors and Cell Signaling, University of Houston, Houston, USA

**Keywords:** Anxiety, ErbB4, Estrogen receptor β, Gut microbiota, Hypothalamic-pituitary-adrenal axis, Inflammatory bowel disease, Stress

## Abstract

**Background:**

Although the lack of estrogen receptor β (ERβ) is a risk factor for the development of inflammatory bowel disease (IBD) and psychiatric disorders, the underlying cellular and molecular mechanisms are not fully understood. Herein, we revealed the role of gut microbiota in the development of IBD and related anxiety-like behavior in ERβ-deficient mice.

**Results:**

In response to dextran sodium sulfate (DSS) insult, the ERβ knockout mice displayed significant shift in α and β diversity in the fecal microbiota composition and demonstrated worsening of colitis and anxiety-like behaviors. In addition, DSS-induced colitis also induced hypothalamic-pituitary-adrenal (HPA) axis hyperactivity in ERβ-deficient mice, which was associated with colitis and anxiety-like behaviors. In addition, RNA sequencing data suggested that ErbB4 might be the target of ERβ that is involved in regulating the HPA axis hyperactivity caused by DSS insult. Gut microbiota remodeling by co-housing showed that both the colitis and anxiety-like behaviors were aggravated in co-housed wild-type mice compared to single-housed wild-type mice. These findings suggest that gut microbiota play a critical role in mediating colitis disease activity and anxiety-like behaviors via aberrant neural processing within the gut-brain axis.

**Conclusions:**

ERβ has the potential to inhibit colitis development and anxiety-like behaviors via remodeling of the gut microbiota, which suggests that ERβ is a promising therapeutic target for the treatment of IBD and related anxiety-like behaviors.

Video Abstract

**Supplementary Information:**

The online version contains supplementary material available at 10.1186/s40168-022-01356-2.

## Introduction

Inflammatory bowel disease (IBD), including Crohn’s disease and ulcerative colitis, is a group of chronic, disabling diseases that cause gastrointestinal tract symptoms [1]. Psychiatric comorbidities, such as anxiety and depression, frequently occur in IBD patients, with up to one-third of patients affected by anxiety and a quarter affected by depression, which is a significant challenge for the optimal physiological and psychological health of patients [2, 3]. IBD and the related psychiatric comorbidities are considered a disorder of the gut-brain axis, through which the gut can regulate brain functions via the nervous system, hypothalamic-pituitary-adrenal (HPA) axis, and immune response. In addition to the effect of disease activity on the severity of anxiety and depression in IBD patients, sex differences also affect the prevalence of psychiatric symptoms [4, 5]. Female IBD patients are more likely than men to have anxiety, depression, and reduced quality of life [3, 6]. However, the interactions among the gut-brain axis, psychiatric comorbidities, and sex differences in IBD are less clearly understood.

Gut microbiota can directly regulate the gut-brain axis. Abnormal gut microbiota profiles, referred to as gut dysbiosis, influence the host physiology through modulating the gut barrier homeostasis and gut inflammation. Gut dysbiosis is one of the major pathogenic factors involved in IBD development [7]. Increasing evidence suggests the frequent occurrence of gut dysbiosis in IBD patients [8, 9]. Remarkably, gut dysbiosis correlates with a wide range of mental health conditions, such as anxiety and depression, via the microbiota**-**gut-brain axis. After receiving fecal microbiota from patients with irritable bowel syndrome, mice showed faster gut motility, gut barrier dysfunction, colon inflammation, and anxiety-like behavior [10]. Kilinçarslan et al. [11] showed that fecal microbiota transplantation from age-matched healthy donors into the intestine of IBD patients alleviated the severity of anxiety, depression, and obsession, as well as gastrointestinal symptoms. It was further revealed that microbiota manipulation, such as using prebiotics or probiotics, can improve gut abnormalities and neurobehavioral deficits in both animals and humans [12, 13]. Therefore, gut dysbiosis is closely linked to gut dysfunction and the related mood disorders in gastrointestinal diseases.

The composition of gut microbiota remains relatively constant throughout the adult life and may be altered under circumstances that subsequently influence the host’s health status [14]. Intriguingly, the different levels of sex hormones, including testosterone and estradiol, are correlated with the diversity and composition of gut microbiota in humans, indicating an association between sex hormones and gut microbiota [15]. Estrogen receptor (ER) β is an important ligand-activated nuclear transcription factor involved in estrogen signaling, and it is the predominant ER subtype in colon tissue where it plays an important role in colonic mucosal homeostasis by maintaining the integrity of tight junctions and the barrier function [16, 17]. Intestinal epithelial cell-specific deletion of ERβ can alter the gut microbiota composition in mice [18]. This was further supported by the markedly reduced expression of ERβ in active ulcerative colitis and Crohn’s disease, suggesting an essential role of ERβ in IBD development [17, 19]. Additionally, accumulating evidence has demonstrated that ERβ could exert anxiolytic effects in rodents. Furthermore, selective ERβ agonists can reduce anxiety-like behaviors in mice [20]. It appears that ERβ is important for maintaining gut homeostasis and the mental health condition. However, the mechanisms underlying the involvement of ERβ in microbiota-mediated IBD development as well as altered behavior remain to be determined.

In this study, we found that ERβ knockout (ERβ^−/−^) mice with dextran sulfate sodium (DSS)-induced acute colitis exhibited anxiety-like behavior. Meanwhile, ERβ deficiency in mice caused alterations in gut microbiota composition and increased the susceptibility of colitis. HPA axis hyperactivity, rather than neuroinflammation, is involved in the IBD-related anxiety-like disorders after loss of ERβ in mice. Co-housing the wild-type (WT) mice with ERβ^−/−^ mice further showed that perturbed gut microbiota are involved in the IBD-related anxiety-like behaviors. Our findings highlight that gut microbiota act as a triggering event in IBD and related anxiety-like behaviors in ERβ-deficient mice.

## Materials and methods

### Mice

Mice were housed in a specific pathogen-free facility of the Third Military Medical University and had ad libitum access to standard mouse chow and water in a controlled condition under a 12-h light-dark cycle. The experimental procedures were performed in line with the Guidelines for Animal Committee of Third Military Medical University, and were approved by the Institutional Review Board (approval no.: AMUWEC20210373). ERβ^−/−^ mice were generated by crossing ERβ^**+/−**^ male with female mice [21]. Transgenic mice were backcrossed with C57BL/6 mice for at least seven generations. The sample sizes for the animal experiments are indicated in the figure legends.

### DSS colitis model

Acute colitis was induced in 9–10-week-old male mice by adding 2% DSS (36,000–50,000 MW; MP Biomedicals, Solon, OH, USA) to the drinking water for 5 days. The severity of colitis was determined daily on the basis of rectal bleeding and diarrhea, and scored as follows [22]: stool bleeding, 0 = normal, 1 = red, 2 = dark red, and 3 = gross bleeding; stool consistency, 0 = normal, 1 = soft, 2 = very soft, and 3 = diarrhea.

### Co-housing experiment

For the co-housing experiments, 4-week-old WT and ERβ^−/−^ male mice from the same breeders were divided into either single-housed (SiHo) or co-housed (CoHo) conditions for 6 weeks. Two or three WT mice were CoHo with an equal number of ERβ^−/−^ mice in one CoHo cage, and four or five mice with same genotype were housed in a single SiHo cage. CoHo mice were compared to their SiHo littermates as controls.

### Study design

#### Study 1

**Fig. 1:**
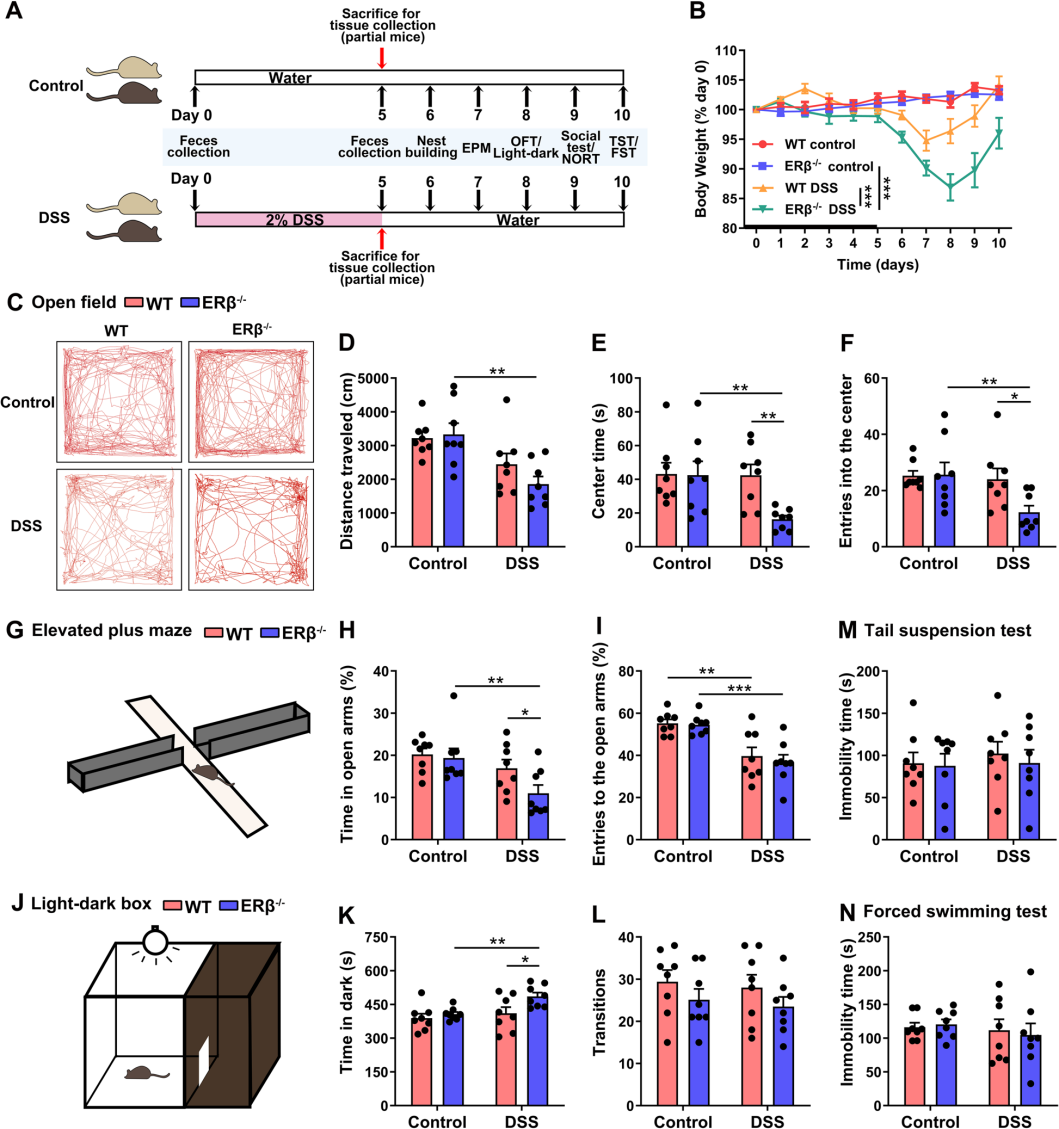
Increased anxiety-like behavior in ERβ^−/−^ mice following the induced experimental colitis, but no significant depressive behavior. **A** Study design: 9–10-week-old WT or ERβ^−/−^ male mice were randomly exposed to normal water or DSS for 5 days. Some mice were sacrificed under anesthesia for tissue collection (brain, colon, and blood) on day 5 after initial DSS exposure. The remaining mice were allowed to recover by drinking normal water for another 5 days, and underwent several behavioral tests. The fecal microbiota were collected on days 0 and 5 after initial DSS exposure. **B** Time evolution of DSS-induced colitis in WT mice. Heavy line: 5-day period of DSS administration. Body weight was recorded. *n* = 9/group. **C–F** Representative trajectory diagrams of four groups of mice in the open field test (**C**), total distance traveled (**D**), time spent in the central zone (**E**), and entries into the center (**F**) of WT and ERβ^−/−^ mice treated with water or DSS. **G–I** Diagram of the elevated plus maze (**G**), percentage of time spent by mice in the open arms (**H**), and frequency of entries into the open arms (**I**). **J–L** Diagram of the light-dark box (**J**), time spent by mice in the dark chamber (**K**), and transitions between the two chambers (**L**) in the light-dark box test. **M, N** The immobility time of mice among the four groups in the tail suspension test (**M**) and forced swimming test (**N**). Data are presented as mean ± SEM. Statistical comparisons were performed by two-way ANOVA. *n* = 8/group. *P < 0.05, **P < 0.01, ***P < 0.001. EPM, elevated plus maze; OFT, open field test; NORT, novel object recognition test; TST, tail suspension test; FST, forced swimming test

In this study, 9–10-week-old WT or ERβ^−/−^ male mice were randomly delegated to exposure to normal water or DSS for 5 days. In total, 8–9 mice per group were sacrificed under anesthesia for tissue collection (brain, colon, and blood) at day 5 after initial exposure to DSS. The remaining mice were allowed to recover by drinking normal water for an additional 5 days. The remaining mice were divided into two batches (eight mice per group in one batch), and a series of behavioral tests were performed on these mice during on days 6–10 following initial DSS exposure. Nest building, elevated plus maze, open field, three-chamber test, and tail suspension test were performed in batch 1, whereas light-dark box, novel object recognition test, and force swimming test were performed in batch 2. The fecal microbiota were collected on days 0 and 5 after initial DSS exposure. The experiment timeline is presented in Fig. 1A.

#### Study 2

In this study, 4-week-old WT and ERβ^−/−^ male mice were SiHo or CoHo for 6 weeks (42 days). Then, the mice were administered with 2% DSS for 5 days. Nine mice per group were sacrificed for tissue collection (brain, colon, and blood) at day 5 after initial DSS exposure. The remaining nine mice per group underwent anxiety-like behavioral tests, including elevated plus maze, open field, and light-dark box on days 7–9 following initial DSS exposure. The fecal microbiota were collected before and 42 days after exposure to the CoHo experiment. The experiment timeline is presented in Fig. 6A.

### Statistical analysis

Data were analyzed using the Statistical Package for the Social Sciences (version 25.0 for Windows; IBM Corp., Armonk, NY, USA). Data were assessed for normal distribution and plotted in the figures as mean ± SEM or box plots. Two-way ANOVA with Boferroni’s post hoc test was used to compare two variables. Relative abundances of specific bacteria among the groups were tested using the non-parametric Wilcoxon rank sum test. For all statistical comparisons, *P < 0.05, **P < 0.01, and ***P < 0.001.

The detailed methods are described in the “Supplementary materials and methods”.

## Results

### DSS-induced colitis led to anxiety, but not depression-like behaviors, in ERβ^−/−^ mice

As shown in Fig. 1B, the general health status of DSS-treated ERβ^−/−^ mice was significantly worsened. After DSS administration for 5 days, the body weight of ERβ^−/−^ mice steadily decreased, and ERβ^−/−^ mice lost 13.1% of their initial body weight by day 8. In contrast, WT mice exhibited minimal body weight loss and recovered body weight rapidly when fed with normal drinking water.

Increasing evidence suggests that anxiety- and depression-like behaviors are common in IBD, but the causality of the link between ERβ and IBD-induced mood and behavior abnormalities has not been proven [23]. To determine whether DSS-induced colitis caused these behaviors, both ERβ^−/−^ and WT male mice were treated with 2% DSS mixed in drinking water for 5 days, followed by a panel of behavioral tests. In the open field test, the groups of mice showed similar locomotor activity (total distance traveled) (Fig. 1C, D). However, in the open field test, DSS-treated ERβ^−/−^ mice exhibited reduced time duration (Fig. 1E) and frequency in the center area (Fig. 1F) compared to control ERβ^−/−^ and DSS-treated WT mice, indicating that DSS treatment increased anxiety-like behavior in ERβ^−/−^ mice. In the elevated plus maze test, DSS-treated ERβ^−/−^ mice spent significantly less time in the open arms and entered the open arms less frequently than control ERβ^−/−^ mice (Fig. 1G–I). Furthermore, there were significant differences in the time spent in the open arms between DSS-treated WT and ERβ^−/−^ mice (Fig. 1H). In the light-dark box test, DSS-treated ERβ^−/−^ mice spent significantly more time exploring in the dark chamber than the control ERβ^−/−^ and DSS-treated WT mice (Fig. 1K). However, no difference was observed in the transition frequencies between light and dark chambers among the four groups (Fig. 1L). These results suggest that ERβ loss leads to an increase in anxiety-like behavior following DSS treatment.

The tail suspension test and forced swimming test were conducted to explore the depression-like behavior in mice. In these two assays, mice from four groups showed indistinguishable immobility time (Fig. 1M, N), indicating no significant depressive behaviors in these mice. In the nest building, Y maze, novel object recognition, and three-chamber tests, there were no significant differences between WT and ERβ^−/−^ mice treated with DSS, suggesting that ERβ deficiency might not influence the sensorimotor function, memory function, and social interactions in mice following DSS treatment (Fig. S1). Collectively, these data suggest that ERβ protect against anxiety in a variety of anxiogenic situations in mice with colitis.

### ERβ deficiency resulted in altered composition of gut microbiota

**Fig. 2:**
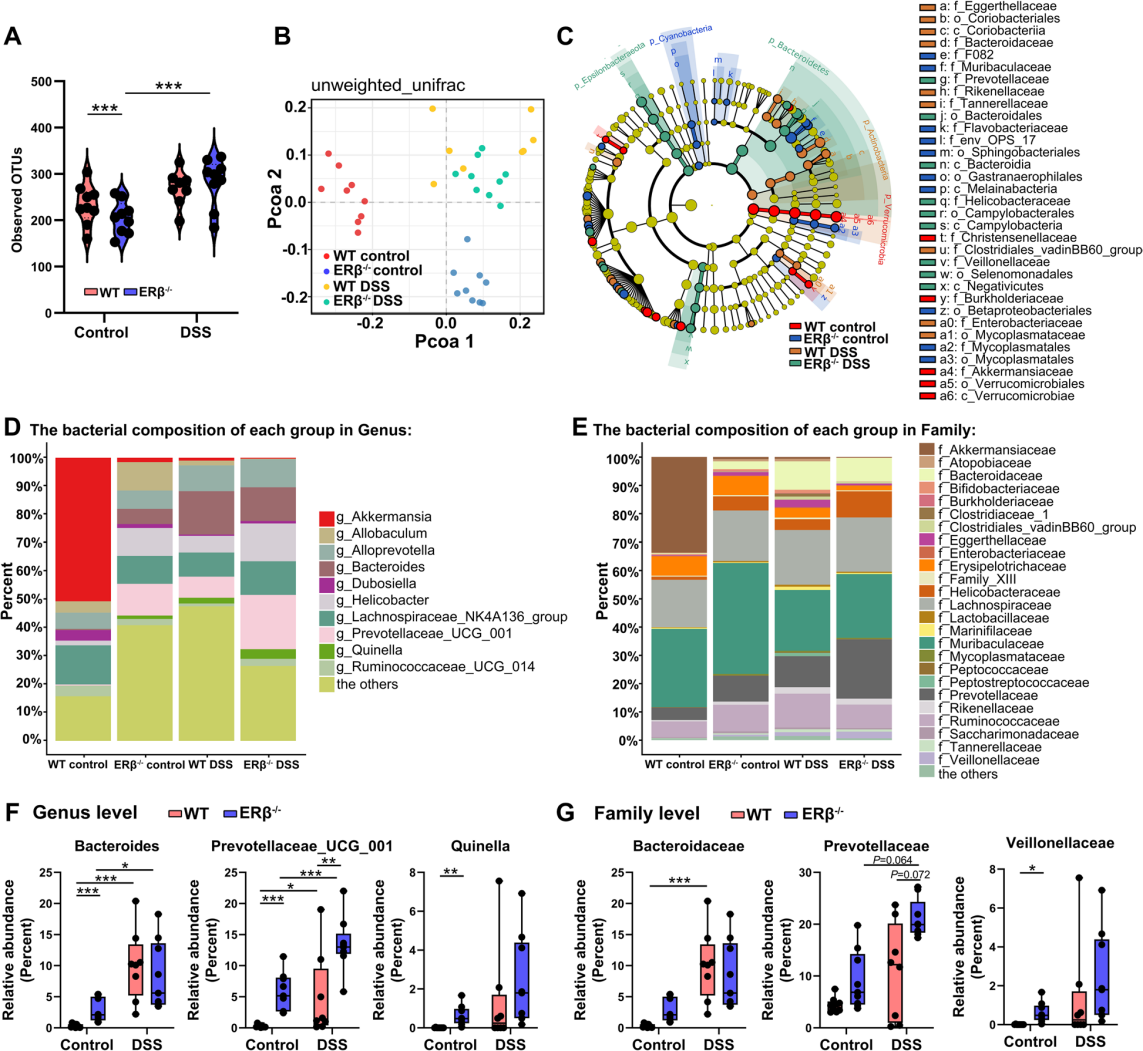
Differences in the fecal microbiota of WT and ERβ^−/−^ mice under baseline and inflammatory states. **A** Community richness calculated by observed OTUs. **B** Principal coordinates analysis of microbial unweighted UniFrac compositional differences. **C** Taxonomic cladogram obtained using LEfSe analysis. **D, E** Bar graph of bacterial abundance at the genus (**D**) and family (**E**) levels. **F, G** Relative abundances of substantially changed bacteria taxa at the genus (**F**) and family (**G**) levels. Data are presented as boxplots. Statistical comparison was performed using non-parametric Wilcoxon rank sum test. *n* = 9/group, except for *n* = 8 in DSS-treated WT group. *P < 0.05, **P < 0.01, ***P < 0.001

Recent studies have confirmed that changes in the gut microbiota are often closely associated with anxiety disorders [24, 25]. To determine whether alteration of gut microbiota is involved in the anxiety-like behavior in DSS-treated ERβ^−/−^ mice, the fecal microbiota composition was analyzed using MiSeq 16S rRNA gene sequencing. ERβ deficiency led to a reduction in community richness (α diversity) at baseline, as shown by the observed operational taxonomic units (OTUs) (Fig. 2A), suggesting that the gut microbiota of control ERβ^−/−^ mice had less species variation compared to control WT mice. After DSS, the observed OTUs were increased in DSS-treated ERβ^−/−^ mice compared to the control ERβ^−/−^ mice (Fig. 2A). β diversity was determined by a principal coordinates analysis (PCoA) plot based on the Bray-Curtis distance and showed separate clusters for ERβ^−/−^ and WT mice under baseline or DSS-induced inflammatory states (Fig. 2B).

ERβ deletion in mice dramatically altered the composition of gut microbiota (Fig. 2C–E). Comparative analysis was performed to identify the taxa with significantly altered relative abundance at various classification levels of bacteria. Compared to control WT mice, control ERβ^−/−^ mice had a higher abundance of bacteria belonging to the genera *Bacteroides*, *Prevotellaceae_UCG_001*, and *Quinella* (Fig. 2F). At the family level, a significantly higher proportion of *Veillonellaceae* was observed in control ERβ^−/−^ mice compared to control WT mice (Fig. 2G). Control ERβ^−/−^ mice also showed enrichments in the classes *Bacteroidia*, *Campylobacteria*, and *Negativicutes*, as well as the order *Bacteroidales* and *Campylobacterales* compared to control WT mice (Fig. S2). Compared to the control WT mice, DSS-treated WT mice exhibited higher levels of genera *Bacteroides* and *Prevotellaceae_UCG_001*, as well as the family *Bacteroidaceae* (Fig. 2F, G). DSS-treated ERβ^−/−^ mice showed higher abundances of *Bacteroides* and *Prevotellaceae_UCG_001* at the genus level compared to the control ERβ^−/−^ mice (Fig. 2F, G). The relative abundance of genus *Prevotellaceae_UCG_001* was higher in ERβ^−/−^ mice than WT mice after DSS treatment, and there was an upward trend in the abundance of the family *Prevotellaceae* in DSS-treated ERβ^−/−^ mice compared to DSS-treated WT mice (Fig. 2F, G).

Next, we investigated the gut microbiota compositions of 2-month-old WT and ERβ^−/−^ female mice under homeostasis conditions (Fig. S3). However, no significant differences were observed in the α and β diversity of gut microbiota composition between WT and ERβ^−/−^ female mice. The gut microbiota composition was similar between the WT and ERβ^−/−^ female mice.

Overall, these data showed that ERβ deficiency induced a shift in the gut microbiota composition under baseline and inflammatory states in male mice.

### ERβ deficiency aggravated the development of DSS-induced colitis in mice

**Fig. 3:**
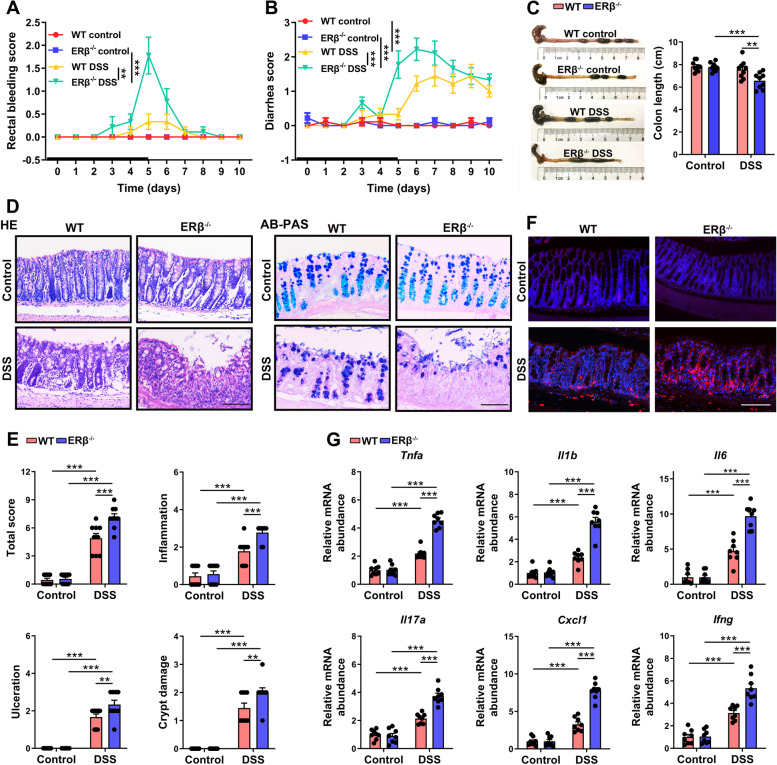
ERβ deficiency aggravated the development of DSS-induced colitis. **A, B** Time evolution of DSS-induced colitis in WT and ERβ^–^^/^^–^ mice. Heavy line: 5-day period of DSS administration. Rectal bleeding score (**A**) and diarrhea score (**B**) were evaluated. *n* = 9/group. **C** Mice were sacrificed on day 5 after DSS treatment to measure the colon length. *n* = 9/group. **D** Histology of distal colon tissues collected at day 5 was examined by hematoxylin and eosin (HE) and Alcian Blue Periodic Acid Schiff (AB-PAS) staining. **E** Composite score of histopathology (inflammation, ulceration, and crypt damage scores). *n* = 9/group. **F** Infiltrated macrophages (F4/80^+^) in the colon stained by immunofluorescence. **G** Quantitative real-time PCR analysis of mRNA expressions of inflammatory cytokines (*Tnfα*, *Il1b*, *Il6*, *Il17s*, *Cxcl1*, and *Ifng*) in whole colon tissues. Scale bars in **D** and **F** = 100 μm. *n* = 8/group. Data are presented as mean ± SEM. Statistical comparisons were performed using two-way ANOVA. *P < 0.05, **P < 0.01, ***P < 0.001

To evaluate the role of ERβ in colitis pathogenesis, rectal bleeding and stool consistency were monitored for 10 days. After DSS administration for 5 days, ERβ^−/−^ mice suffered from significant rectal bleeding (Fig. 3A) and diarrhea (Fig. 3B).

The colon lengths were comparable between different genotypes under normal conditions, while on day 5 post-DSS initiation, the colon length was significantly shortened in ERβ^−/−^ mice than WT mice (Fig. 3C). Next, we examined the histological features of colonic tissues with hematoxylin and eosin (HE), Alcian Blue Periodic Acid Schiff (AB-PAS), and tight junction protein (occludin and zonula occludens 1 [ZO-1]) staining. Although ERβ^−/−^ and WT control mice exhibited comparable histological features (Fig. 3D and S4), more severe colonic ulceration, crypt damage, and inflammation were observed in DSS-treated ERβ^−/−^ mice compared to DSS-treated WT mice on day 5 (Fig. 3D,E and Fig. S4), suggesting that ERβ plays a pivotal role in maintaining colonic epithelial homeostasis after DSS-induced gut inflammation. The colon length and histological features of colonic tissues using HE, AB-PAS, and tight junction protein (occludin and ZO-1) staining were evaluated on day 10 after initial DSS exposure. Significantly shorter colon length and more severe colonic ulceration, crypt damage, and inflammation were observed in DSS-treated ERβ^−/−^ mice compared to DSS-treated WT mice on day 10 (Figs. S5 and S6).

The gut inflammatory response is an important mediator between the gut microbiota and brain [26, 27]. Macrophage infiltration and higher levels of pro-inflammatory factors characterize the colonic inflammation in IBD. Immunofluorescence staining showed an increase in infiltrated macrophages (F4/80+ cells) in the colon of DSS-treated ERβ^−/−^ mice compared to DSS-treated WT mice on day 5 (Fig. 3F). We also detected significantly increased mRNA levels of pro-inflammatory cytokine genes in the colon of ERβ^−/−^ mice 5 days post-DSS, including *tumor necrosis factor alpha* (*Tnfa*), *interleukin* (*Il) 1b*, *Il6*, *Il17a*, *C-X-C motif chemokine ligand 1* (*Cxcl1)*, and *interferon gamma* (*Ifng)* (Fig. 3G). Collectively, these data suggest that ERβ^−/−^ mice displayed deficiency of colonic epithelium and robust colitis after DSS treatment.

### HPA axis was dysregulated in ERβ^−/−^ mice with DSS-induced colitis

Previous studies reported that dysbiosis or gut inflammation might induce neurological disorders via neuroinflammation or the HPA axis [24, 28–31]. To confirm whether neuroinflammation is involved in the gut-brain communication in DSS-induced anxiety disorders after ERβ loss, the number of microglia in the brain regions related to anxiety-like behavior was evaluated. There were no significant differences in the number of ionized calcium-binding adapter molecule 1 (Iba1)-positive cells between DSS-treated WT and ERβ^−/−^ mice in the medial prefrontal cortex (mPFC), amygdala, and ventral and dorsal hippocampus post-DSS (Fig. S7).

**Fig. 4:**
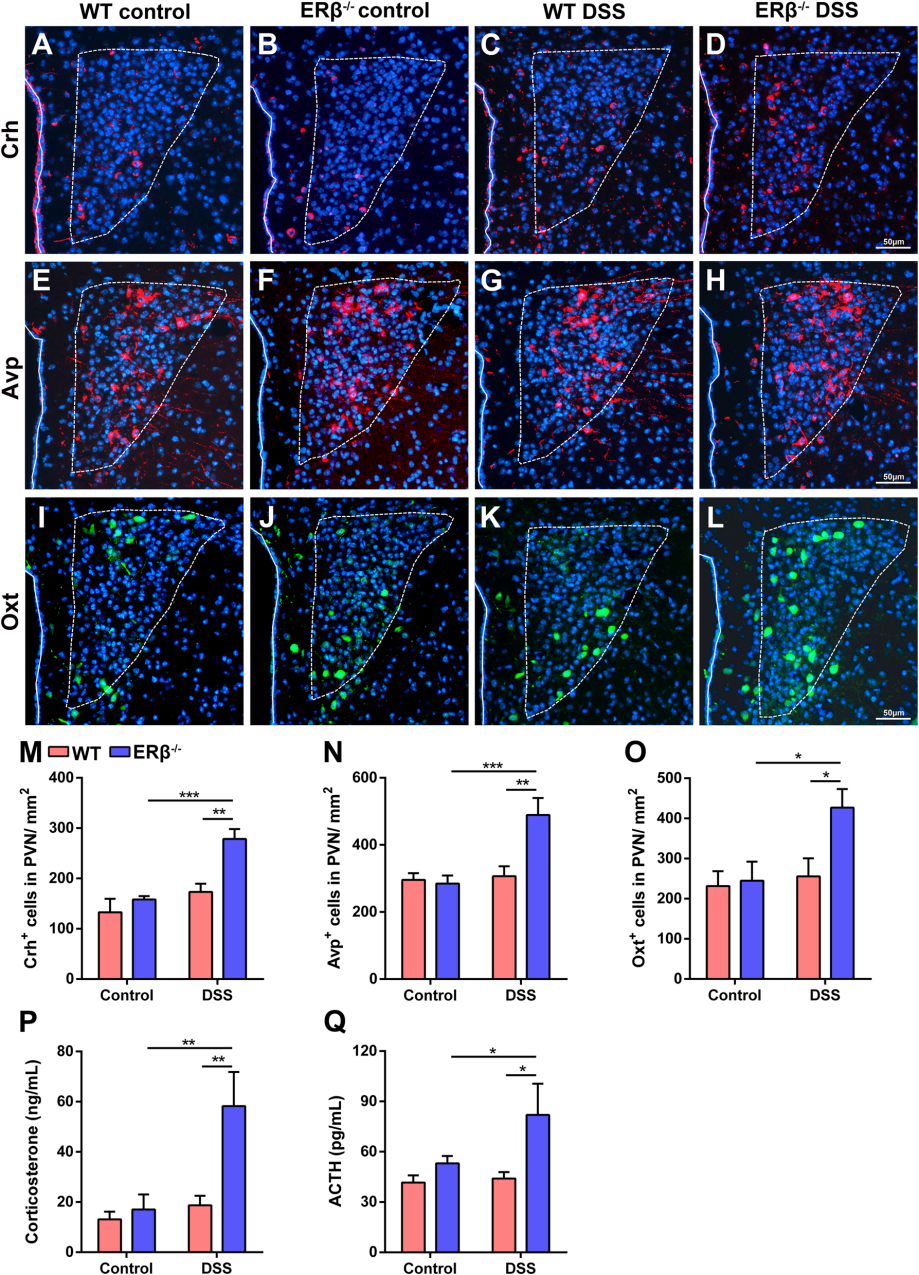
Dysregulated hypothalamic-pituitary-adrenal axis in ERβ^−/−^ mice with DSS-induced colitis. **A–O** Representative images and quantitative analysis of Crh (**A**–**D**, **M**), Avp (**E**–**H**, **N**), and Oxt (**I**–**L**, **O**) immunofluorescence staining within the PVN of WT and ERβ^−/−^ mice under homeostatic conditions and after 5 days of DSS treatment. Scale bars = 50 μm. *n* = 5/group. **P, Q** Plasma corticosterone (**P**) and ACTH (**Q**) levels in baseline conditions and 5 days after DSS treatment in WT and ERβ^−/−^ mice. *n* = 8/group. Data are presented as mean ± SEM. Statistical comparisons were performed using two-way ANOVA. *P < 0.05, **P < 0.01, ***P < 0.001. Crh, corticotropin-releasing hormone; Avp, arginine vasopressin; Oxt, oxytocin; PVN, paraventricular nucleus; ACTH, adrenocorticotropic hormone

The HPA axis is a pivotal component in gut-brain communication and allows the gut to influence mood, such as by causing anxiety [24]. We found that the numbers of corticotropin-releasing hormone (Crh)-positive and arginine vasopressin (Avp)-positive cells were comparable in the paraventricular nucleus (PVN) between WT and ERβ^−/−^ control mice (Fig. 4A–H, M, N). The numbers of Crh-positive and Avp-positive cells in the PVN were significantly increased in the ERβ^−/−^ mice on day 5 post-DSS compared to DSS-treated WT mice (Fig. 4A–H, M, N). Oxytocin (Oxt) also mediates the regulation of HPA axis activity [32]. DSS-treated ERβ^−/−^ mice showed a higher number of Oxt-positive cells in the PVN than control ERβ^−/−^ or DSS-treated WT mice on day 5 post-DSS (Fig. 4I–L, O).

The co-release of Crh and Avp potentiates the release of corticosterone and adrenocorticotropic hormone (ACTH) in response to stressors [33]. Corticosterone and ACTH levels were assessed using enzyme-linked immunosorbent assay, which showed that DSS-treated ERβ^−/−^ mice had elevated plasma corticosterone and ACTH levels compared to DSS-treated WT mice and control ERβ^−/−^ mice 5 days after DSS exposure (Fig. 4P, Q). Overall, ERβ deletion led to increased HPA responsiveness following DSS treatment, indicating that HPA axis hyperactivity exerts a critical role in anxiety-like behaviors observed in DSS-treated ERβ^−/−^ mice.

### ErbB4 (Erb-b2 receptor tyrosine kinase 4) was downregulated in the hypothalamus of ERβ^−/−^ mice with experimental colitis

**Fig. 5:**
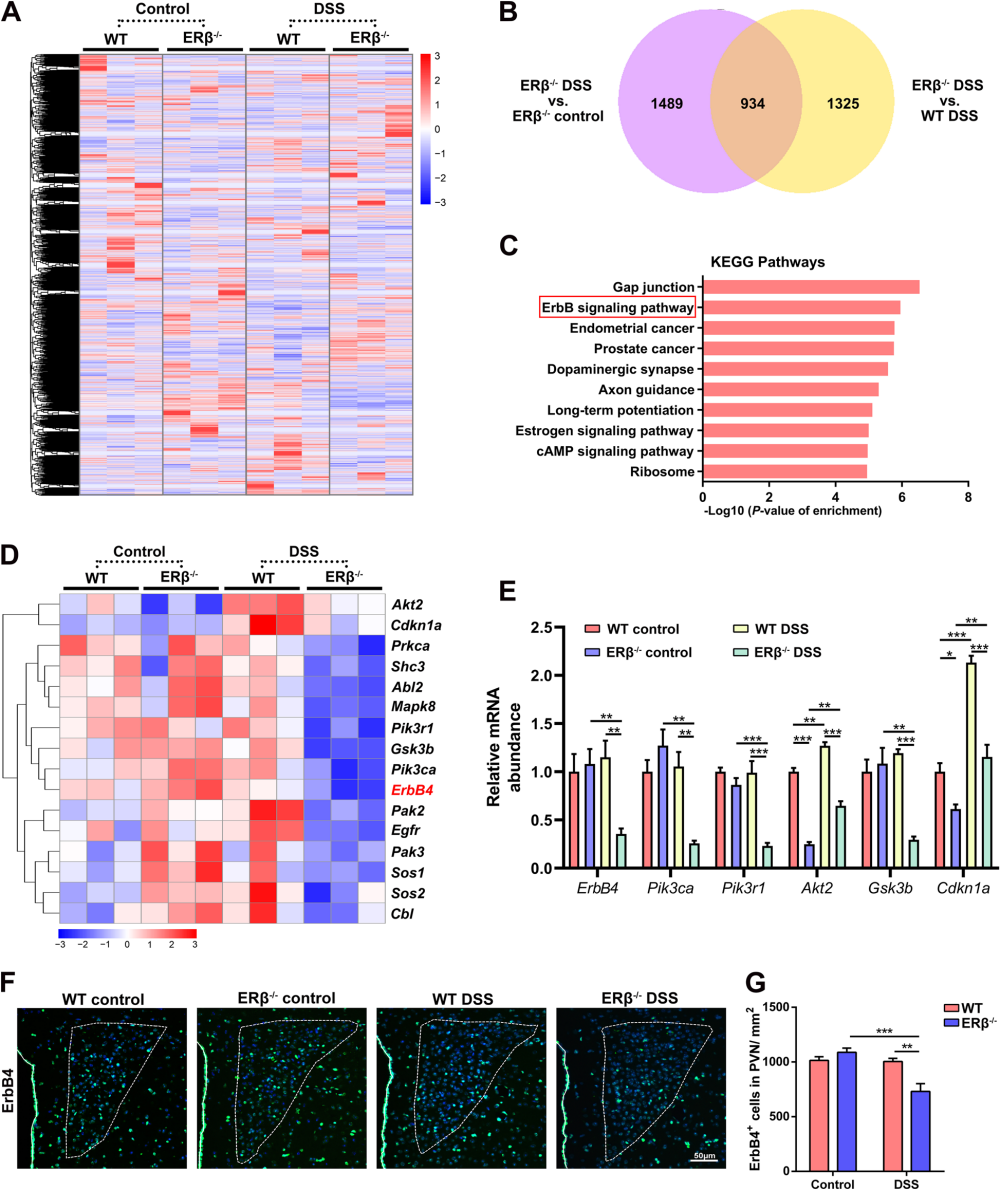
ErbB4 is downregulated in ERβ^−/−^ mice with experimental colitis. **A** Hierarchical clustering heatmap of gene expression profile in hypothalamus of WT and ERβ^−/−^ mice under homeostasis conditions and DSS treatment. *n* = 3/group. **B** Venn diagram of differentially expressed genes (DEGs) between control ERβ^−/−^ and DSS ERβ^−/−^ mice, and between DSS ERβ^−/−^ and DSS WT mice. *n* = 3/group. **C** Signaling pathway enrichment analysis was performed using Kyoto Encyclopedia of Genes and Genomes (KEGG). Top 10 significantly enriched pathways in the hypothalamus related to the 934 overlapping genes plotted by enrichment score. **D** Hierarchical clustering heatmap of gene expression profile of ErbB signaling pathway. *n* = 3/group. **E** Quantitative real-time PCR analysis of mRNA expressions of *ErbB4* and its several downstream genes (*Pik3ca*, *Pik3r1*, *Akt2*, *Gsk3b*, and *Cdkn1a*) in the hypothalamus. *n* = 3/group. **F, G** Representative images (**F**) and quantitative analysis (**G**) of ErbB4 immunofluorescence staining within the paraventricular nucleus (PVN) of WT and ERβ^−/−^ mice in the baseline state and 5 days after onset of DSS exposure. Scale bar = 50 μm. *n* = 5/group. Data are presented as mean ± SEM. Statistical comparisons were performed using two-way ANOVA. *P < 0.05, **P < 0.01, ***P < 0.001

To identify the potential molecular mechanisms underlying the increased anxiety-like behaviors in ERβ-deficient mice with colitis, we performed RNA sequencing (RNA-seq) analysis of the hypothalamus from WT and ERβ^−/−^ mice on day 5. The gene expression profile in the hypothalamus of control WT, control ERβ^−/−^, DSS-treated WT, and DSS-treated ERβ^−/−^ mice are presented in a hierarchical clustered heatmap (Fig. 5A). High expression levels of several hypothalamic neuropeptides were found in DSS-treated ERβ^−/−^ mice (Fig. S8A), which confirms the role of hypothalamus in the anxiety disorder caused by ERβ^−/−^ deficiency during colitis. A Venn diagram showed that there were 1489 DSS-regulated differently expressed genes (DEGs) between control ERβ^−/−^ mice and DSS-treated ERβ^−/−^ mice, 1325 ERβ-regulated DEGs between DSS-treated ERβ^−/−^ and DSS-treated WT, and 934 genes that were co-regulated by DSS and ERβ (Fig. 5B). In addition, hierarchical clustering analysis of the 934 co-regulated genes showed that ERβ deletion increased the effect of DSS on the co-regulated genes (Fig. S8B).

ErbB4 is involved in regulating various neuropsychiatric disorders, including schizophrenia [34], anxiety [35], and seizures [36]; this receptor is mainly expressed in the PVN of hypothalamus [37]. Accordingly, the ErbB signaling pathway was found to have significant enrichment in the Kyoto Encyclopedia of Genes and Genomes (KEGG) pathways (Fig. 5C, D). The gene expressions of *ErbB4* and its several downstream genes, namely, *phosphatidylinositol-4,5-bisphosphate 3-kinase catalytic subunit alpha* (*Pik3ca*), *phosphoinositide-3-kinase regulatory subunit 1* (*Pik3r1*), *AKT serine/threonine kinase 2* (*Akt2*), *glycogen synthase kinase 3 beta* (*Gsk3b*), and *cyclin-dependent kinase inhibitor 1a* (*Cdkna1a*), in the hypothalamus were further identified by quantitative real-time PCR. Decreased mRNA levels of *ErbB4*, *PIK3ca*, *PIK3r1*, *AKT2*, *GSK3b*, and *CDKN1a* were shown in DSS-treated ERβ^−/−^ mice compared to DSS-treated WT mice (Fig. 5E). Next, we performed immunofluorescence staining to verify the protein expression level of ErbB4 in the hypothalamus (Fig. 5F, G). The number of ErbB4-positive cells in the hypothalamic PVN of control ERβ^−/−^ mice was comparable to that in control WT mice (Fig. 5F, G). However, the number of ErbB4-positive cells in the PVN of DSS-treated ERβ^−/−^ mice was significantly reduced compared to that in the DSS-treated WT mice on day 5 post-DSS (Fig. 5F, G). Given the decreased ErbB4 expression in the hypothalamus of ERβ^−/−^ mice in response to DSS, as well as its established role in regulating brain functions, we hypothesized that ErbB4 may be an important regulator of anxiety-like behavior in ERβ^−/−^ mice with colitis.

### Gut microbiota of ERβ^−/−^ mice were sufficient to facilitate DSS-induced colitis and anxiety-like behaviors

**Fig. 6:**
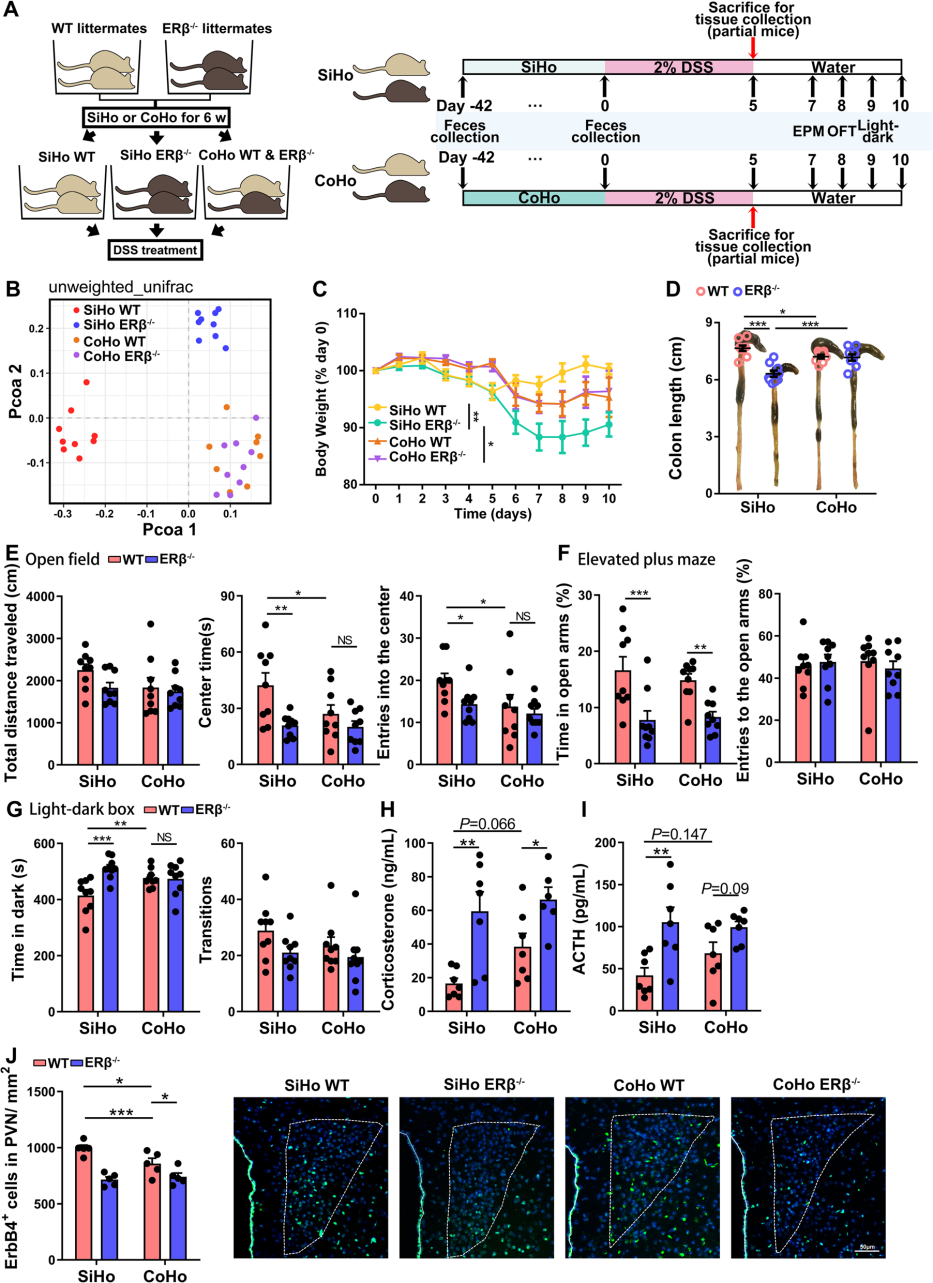
WT mice co-housed with ERβ^−/−^ mice display aggravated colitis and increased anxiety-like behavior. **A** Schematic representation and study design of the co-housing and DSS treatment of WT and ERβ^−/−^ mice. In this experiment, 4-week-old WT and ERβ^−/−^ male mice were single-housed (SiHo) or co-housed (CoHo) for 6 weeks (42 days). Then, the mice were administered with 2% DSS for 5 days. Some of the mice were sacrificed for tissue collection (brain, colon, and blood) on day 5. The remaining mice were exposed to anxiety-related behavioral tests. The fecal microbiota were collected before and 42 days after exposure to the CoHo experiment. **B** Principal coordinates analysis of microbial unweighted UniFrac compositional differences among SiHo WT, SiHo ERβ^−^^/^^−^, CoHo WT and CoHo ERβ^−/−^ mice before DSS treatment. *n* = 9/group, except for *n* = 8 in the CoHo WT group. **C** Body weight of SiHo WT, SiHo ERβ^−^^/^^−^, CoHo WT and CoHo ERβ^−/−^ mice at various times after DSS treatment. *n* = 9/group. **D** Colon length of SiHo WT, SiHo ERβ^−^^/^^−^, CoHo WT and CoHo ERβ^−/−^ mice on day 5 after DSS treatment. *n* = 9/group. **E** Total distance traveled, time spent in the center, and entries into the center in the open field test. *n* = 9/group. **F** Percentage of time spent in the open arms and percentage of entries into the open arms in the elevated plus maze test. *n* = 9/group. **G** Time in the dark and total transitions in the light-dark box test. *n* = 9/group. **H, I** Plasma corticosterone (**H**) and ACTH (**I**) levels in SiHo WT, SiHo ERβ^−^^/^^−^, CoHo WT and CoHo ERβ^−/−^ mice 5 days after DSS treatment. *n* = 7/group, except for *n* = 6 in the CoHo ERβ^−/−^ group for plasma corticosterone index. **J** Quantitative analysis and representative images of ErbB4 immunofluorescence staining within the paraventricular nucleus (PVN) of SiHo WT, SiHo ERβ^−^^/^^−^, CoHo WT and CoHo ERβ^−/−^ mice 5 days after DSS exposure. Scale bar = 50 μm. *n* = 5/group. Data are presented as mean ± SEM. Statistical comparisons were performed by two-way ANOVA. *P < 0.05, **P < 0.01, ***P < 0.001. EPM, elevated plus maze; OFT, open field test

To explore whether DSS-induced colitis severity and anxiety-like behaviors in ERβ^−/−^ mice correlated with changes in gut microbiota, the microbiota transfer studies by CoHo of WT and ERβ^−/−^ mice were performed, in which mice were exposed to the microbiota of other mice based on their coprophagia (Fig. 6A). The β diversity results are presented using the PCoA plot, which revealed an equilibrated gut microbial landscape in CoHo mice before DSS treatment (Fig. 6B). The microbiota dissimilarity between SiHo and CoHo WT mice was similar to that of SiHo WT vs. SiHo ERβ^−/−^ mice (Fig. S9A, calculated from Fig. 6B), which indicated that WT mice CoHo with ERβ^−/−^ mice developed a similar microbiota composition to the ERβ^−/−^ mice. Meanwhile, the microbiota dissimilarity between SiHo and CoHo WT mice was significantly different from that between CoHo WT and CoHo ERβ^−/−^ mice (Fig. S9A, calculated from Fig. 6B), suggesting that ERβ^−/−^ mice CoHo with WT mice developed a microbiota composition that was significantly different from WT mice. The PCoA plot and dissimilarity data revealed that the gut microbiota composition of CoHo WT and CoHo ERβ^−/−^ mice were more similar to SiHo ERβ^−/−^ mice than the SiHo WT mice. We further assessed the effects of CoHo on the abundance of specific bacteria before DSS treatment. The abundances of genera *Bacteroides* and *Prevotellaceae_UGC_001*, and the families *Bacteroidaceae*, *Prevotellaceae*, and *Veillonellaceae* were similar in CoHo WT and CoHo ERβ^−/−^ mice (Fig. S9B–D).

Next, we evaluated the colitis severity, anxiety-like behaviors, levels of stress-related hormones, and expression of ErbB4 in SiHo WT mice, SiHo ERβ^−/−^ mice, CoHo WT, and CoHo ERβ^−/−^ mice after DSS treatment. In line with the aforementioned results, SiHo ERβ^−/−^ mice were more susceptible to DSS-induced colitis (Fig. 6C, D) and related anxiety-like behaviors compared to SiHo WT mice (Fig. 6E–G). Of note, based on the altered gut microbiota in CoHo mice, there was a significant difference in the clinical activity-related colitis. Compared to SiHo WT mice, CoHo WT mice showed a tendency toward lower body weight (Fig. 6C), worse colitis severity (Fig. 6D), and more severe anxiety-like behaviors (less time and frequency in the center area of the open field, and more time in the dark side of the light-dark box) (Fig. 6E–G).

Furthermore, the plasma levels of corticosterone and ACTH showed a trend toward higher levels in CoHo WT mice compared to SiHo WT mice (Fig. 6H, I). Immunofluorescence staining showed that the number of ErbB4-positive cells was significantly reduced in the PVN of CoHo WT mice compared to SiHo WT mice after DSS treatment (Fig. 6J).

The aforementioned evidence suggests that gut microbiota dysbiosis contributes to the higher colitis severity and related anxiety-like behaviors in ERβ^−/−^ mice. In addition, decreased hypothalamic ErbB4 expression might mediate the increased HPA axis activity and anxiety in ERβ-deficient mice compared to WT mice during visceral stress from the gut.

## Discussion

In this study, we provide evidence that ERβ deficiency results in multiple behavioral abnormalities indicative of anxiety, impaired gut microbiota composition in mice, and increased susceptibility to DSS-induced colitis. We showed that highly coordinated differential gene expressions in the hypothalamus, a key brain region involved in anxiety and stress, may contribute to the elicited anxiety-like behavior of DSS-treated ERβ^−/−^ mice. In addition, more severe colitis and anxiety-like behaviors were detected in WT mice with DSS-induced colitis when the gut microbial landscape was equilibrated by CoHo with ERβ^−^^/^^−^ mice, thereby unequivocally proving that gut microbiota are responsible for the deleterious effects on the gut and behavior of ERβ-deficient mice with colitis.

The gut-brain axis is a bidirectional communication network that connects the enteric and central nervous systems via the HPA axis, immune responses, and nervous system. Recent studies have revealed the crucial role of the gut-brain axis in IBD progression. IBD is a chronic and devastating gastrointestinal disease, which is commonly complicated by psychological comorbidities. It has been reported that up to 60–80% of IBD patients with active disease and 30% with clinical remission suffer from mood disorders, especially anxiety and depression [38]. Therefore, recently, researchers have explored the idea of an integrated model of care for both psychological and physiological disorders in IBD patients [39]. However, the connections between mental disorders and IBD are complex and still unclear. An increasing number of studies have focused on the contributions of sex hormones on the susceptibility toward and severity of IBD; these studies have shown that the age of IBD onset varies with sex [40]. A cross-sectional cohort study reported that women with IBD showed significant changes in symptom severity during periods of hormonal fluctuation, including menstruation, pregnancy, post-partum, and post-menopause [41]. Furthermore, the prevalence of anxiety and depression was higher in women with IBD than men with IBD [3]. ERβ is one of the most important estrogen receptors that mediate the sex hormone effects. In the present study, we found that ERβ deficiency caused elevated anxiety-like behaviors in DSS-treated mice, but did not affect depression-like behaviors, sensorimotor function, memory function, or social interaction. Our findings suggest that ERβ is a protective gene that confers resilience against anxiety in mice with colitis. The susceptibility to anxiety disorders has also been shown in other models with ERβ deficiency [42]. However, the underlying mechanisms are still not fully understood.

A growing body of evidence indicates that gut microbiota are involved in mental health [43, 44]. We found that the fecal microbiota community richness (α diversity) and microbiota composition (β diversity) were significantly altered either under baseline conditions or an inflammatory state. The control ERβ^−/−^ mice displayed enrichment of *Bacteroides*, *Prevotellaceae_UCG_001*, and *Quinella* at the genus level, and *Veillonellaceae* at the family level, compared to the control WT mice. Furthermore, the abundance of *Prevotellaceae_UCG_001* was higher in DSS-treated ERβ^−/−^ mice than DSS-treated WT mice. Chen and Jiang et al. [45, 46] found that the abundance of *Bacteroides* was positively associated with anxiety severity in patients with generalized anxiety disorder. Patients with comorbid inflammatory bowel syndrome and anxiety/depression also had higher abundances of *Prevotella/Prevotellaceae* and *Bacteroides* [47]. *Bacteroides* and *Prevotellaceae_UCG_001* might be the key bacteria that regulate anxiety disorder in ERβ^−/−^ mice with colitis. However, the study also found that the α and β diversity were similar between adult female WT and ERβ^−/−^ mice under homeostasis conditions. Importantly, there might be sex differences in the effects of ERβ on gut microbiota modulation, which need further investigation.

Gut inflammatory responses are critically involved in interactions in the microbiota-gut-brain axis and the pathophysiology of anxiety disorder [26, 27]. Jang et al. [48] found that dysbiosis caused by ampicillin could induce gastrointestinal inflammation, which, in turn, may result in anxiety-like behaviors in mice. De Palma et al. [10] demonstrated that transplantation of fecal microbiota from inflammatory bowel syndrome patients altered the expression of inflammation-related genes in colonic tissues, and caused anxiety-like behaviors in the recipient mice. In the present study, ERβ^−/−^ mice exposed to DSS exhibited signs of colitis, more severe colonic ulceration, crypt damage, and inflammation compared to DSS-treated WT mice. The dysbiosis induced by ERβ deficiency might contribute to the elevated levels of colon inflammation. *Prevotellaceae*, *Bacteroidaceae*, and *Veillonellaceae* families are involved in the IBD pathogenesis by interacting with host genetics. Mice with G protein-coupled receptor 109a and recombination activating gene 1 knockout showed spontaneous rectal prolapse and colonic inflammation and had increased abundances of *Bacteroidaceae* and *Prevotellaceae* in the colon [49]. Na+/H+ exchanger 3 (NHE3) knockout mice were characterized by increased abundance of *Bacteroidaceae*, and the onset and severity of experimental colitis were aggravated in the recipient mice who received the microbiota of NHE3^−/−^ mice [50]. Members of the *Veillonellaceae* family are associated with many chronic inflammatory diseases, including IBD, and its abundance was increased in NLR family member x1 (Nlrx1) knockout mice and WT mice CoHo with Nlrx1^−/−^ mice, which worsened the DSS-induced colitis [51].

Previous studies have indicated that the inflammatory responses and HPA axis hyperactivity are plausible mechanisms that explain the behavior alterations induced by gut dysbiosis [29, 48, 52]. Amygdala, hypothalamus, mPFC, and hippocampus are vital brain regions related to anxiety disorders. In this study, we found that there was no significant difference in neuroinflammation and microglia cell density in mPFC, amygdala, and hippocampus of DSS-treated WT mice and DSS-treated ERβ^−/−^ mice. These results indicate that neuroinflammation is not the main cause of the anxiety-like behaviors in DSS-treated ERβ^−/−^ mice. The HPA axis is an important component of the hormonal system that responds to various internal and external stressors. These stressors promote Crh and Avp secretion from the hypothalamus and stimulate ACTH secretion from the pituitary gland, which leads to corticosterone release. A healthy stress response is characterized by a rapid rise in corticosterone levels, followed by a rapid decline with the termination of the stressor, modulated through negative feedback loops. The cumulative stress can increase the corticosterone level and dysfunction of the negative feedback loops, resulting in the development of neuropsychiatric disorders, including anxiety [38]. We observed that the plasma corticosterone and ACTH levels were elevated in ERβ^−/−^ mice after DSS treatment. The hypothalamus is considered the starting point of the HPA axis. The numbers of Crh-, AVP-, and Oxt-positive cells in the PVN were also increased in ERβ^−/−^ mice after DSS treatment. Our data suggest that changes in the HPA axis, rather than neuroinflammation, are involved in the mechanism underlying dysbiosis caused by ERβ deficiency, which influences anxiety-like behaviors.

The transcriptome data suggest that the ErbB pathway, especially the ErbB4 pathway, is downregulated in the hypothalamus of ERβ^−/−^ mice following DSS treatment compared to DSS-treated WT mice. Moreover, both the mRNA expression of ErbB4 in the hypothalamus and the number of ErbB4-positive cells in the PVN were significantly reduced in the DSS-treated ERβ^−/−^ mice. It has been confirmed that ErbB4 is highly expressed in the hypothalamus, especially the PVN [37]. Remarkably, alterations in the ErbB4 expression in the amygdala [53, 54] and white matter [55] could induce anxiety-like behaviors in mice. It is important to note that activated neuregulin 1/ErbB4 signaling could partly normalize the stress-induced behavioral changes in rats [56]. These evidence suggest that ErbB4 might be an important regulator of HPA axis activation and progression of anxiety disorders in ERβ-deficient mice treated with DSS.

Numerous studies have indicated that colitis and anxiety-like behaviors could be significantly influenced by transmissible microbial compositions arising from diet changes or host genetic defects [22, 57, 58]. After we transferred the fecal microbiota from ERβ^−/−^ mice to WT mice by CoHo, the gut microbiota composition of CoHo WT mice was almost comparable to that in ERβ^−/−^ mice. There is a growing body of evidence that suggests that the shifts in gut microbiota composition influence anxiety-like behaviors through the microbiota-gut-brain axis [59]. In agreement with these findings, we found that the colonization of WT mice with gut microbiota from ERβ^−/−^ mice using CoHo is sufficient to induce anxiety-like behaviors in mice following DSS treatment. Moreover, the CoHo WT mice exhibited more severe colitis compared to SiHo WT mice. These results confirm the harmful nature of ERβ deficiency in terms of shifts in gut microbiota composition, and the protective effects of ERβ on microbial symbiosis and reduced susceptibility toward colitis and the related anxiety-like behavior. Compared to DSS-treated SiHo WT mice, DSS-treated CoHo WT mice showed a trend toward increased plasma corticosterone level and reduced ErbB4 expression in the hypothalamus, indicating that gut microbiota alteration might be involved in the HPA axis hyperactivity and the anxiety-like behaviors through regulating the ErbB4 expression in the hypothalamus. However, we found that CoHo ERβ^−/−^ mice did not show improvements in anxiety disorders compared to SiHo ERβ^−/−^ mice. This might be because the CoHo experiment can only transfer the gut microbiota among mice, but not eliminate the pre-existing harmful bacteria. Most importantly, the gut microbiota composition of CoHo WT and CoHo ERβ^−/−^ mice were more similar to SiHo ERβ^−/−^ mice than SiHo WT mice, as shown by the β diversity. This evidence indicates that pretreatment with antibiotics might be necessary for the treatment of IBD patients who have comorbid anxiety or depression before targeting gut microbiota modulation (such as fecal transplantation, probiotics, and prebiotics).

## Conclusions

In summary, we found that gut dysbiosis induced by ERβ deficiency is crucial for the development of IBD and anxiety-like behavior by regulating the HPA axis hyperactivity. Downregulation of ErbB4 in the hypothalamus is a potential mechanism underlying the HPA axis hyperactivity. Our findings highlight the novel role of ERβ in gut-brain communications, which might be one of the reasons for sex differences in IBD and the related psychiatric comorbidities. This study also provides a possible therapeutic approach for psychiatric comorbidities in IBD.

## Supplementary Information

**Additional file 1: Table S1.** Scoring system for histological changes in the colon. **Table S2.** The sequences of primers used in this study. **Figure S1.** ERβ deficiency did not influence the sensorimotor function, memory function, or social interactions in mice following induced experimental colitis. **(A)** Nest score in the nest building test was performed among the four groups to detect the sensorimotor functions. **(B)** Spatial memory was assessed by percentage of spontaneous alterations in the Y maze test. **(C)** Recognition memory was detected by the discrimination index in the novel object recognition test. **(D, E)** Time spent in each chamber and time spent in sniffing a novel mouse or novel object were used to test the sociability in the social approach period (D). Social recognition was evaluated by the time spent in each chamber and time sniffing familiar mouse or novel mouse in social novelty period (E). Data are presented as mean ± SEM. Statistical comparisons were performed by two-way ANOVA or paired *t*-test for the three-chamber test. *n* = 8/group. *P < 0.05, **P < 0.01. **Figure S2.** Fecal microbiota of WT and ERβ^−/−^ mice under baseline and inflammatory states at the class and order levels. **(A)** Bar graph of bacterial abundance at the class level. **(B)** Relative abundances of substantially changed bacterial taxa at the class level. **(C)** Bar graph of bacterial abundances at the order level. **(D)** Relative abundances of substantially changed bacterial taxa at the order level. Data are presented as boxplots. Statistical comparisons were performed using the non-parametric Wilcoxon rank sum test. *n* = 9/group, except for *n* = 8 in the WT DSS group. *P < 0.05. **Figure S3.** ERβ deficiency does not influence gut microbiota composition in adult female mice. **(A)** Community richness calculated by observed OTUs. **(B, C)** Principal coordinates analysis of microbial unweighted UniFrac compositional differences (B), quantified by UniFrac distance (C) between WT and ERβ^−/−^ female mice. **(D)** Taxonomic cladogram obtained using LEfSe analysis. **(E–G)** Bar graph of bacterial abundances at the phylum (E), family (F), and genus (G) levels. Data are presented as boxplots. Statistical comparisons were performed using the non-parametric Wilcoxon rank sum test. *n* = 5/group. **Figure S4.** Tight junctions in WT and ERβ^−/−^ mice under the baseline and inflammatory states on day 5 post-DSS treatment. **(A)** Representative images of immunofluorescence staining for tight junction proteins (occludin and ZO-1) in the distal colon of WT and ERβ^−/−^ mice under homeostatic conditions and 5 days following DSS treatment. Scale bar = 100 μm. **(B–C)** Quantitative real-time PCR analysis of mRNA expressions of occludin and ZO-1 in whole colon tissues of WT and ERβ^−/−^ male mice under homeostatic conditions and 5 days following DSS treatment. *n* = 7-8/group. Data are presented as mean ± SEM. *P < 0.05, **P < 0.01. **Figure S5.** ERβ deficiency aggravated the development of DSS-induced colitis on day 10 after initial DSS exposure. **(A, B)** Mice were sacrificed on day 10 after DSS treatment to measure the colon length. *n* = 7/group. **(C)** Histology of distal colon tissues collected at day 10 was examined by hematoxylin and eosin (HE) and Alcian Blue Periodic Acid Schiff (AB-PAS) staining. Scale bars = 100 μm. **(D–G)** Composite score of histopathology (inflammation, ulceration, and crypt damage scores). *n* = 7/group. *P < 0.05, **P < 0.01, ***P < 0.001. **Figure S6.** Tight junctions in WT and ERβ^−/−^ mice under baseline and inflammatory states on day 10 post-DSS treatment. **(A)** Tight junctions and villi in the colonic epithelium were examined under an electron microscope (scale bar = 2 or 1 μm as indicated in figure), and representative images of immunofluorescence staining (scale bars = 100 μm) of tight junction proteins (occludin and ZO-1) in the distal colon of WT and ERβ^−/−^ mice under homeostasis conditions and day 10 following DSS treatment. **(B–C)** Quantitative real-time PCR analysis of mRNA expressions of occludin and ZO-1 in whole colon tissues of WT and ERβ^−/−^ male mice under homeostatic conditions and 10 days following DSS treatment. *n* = 9/group. Data are presented as mean ± SEM. *P < 0.05, **P < 0.01. **Figure S7.** ERβ deficiency did not significantly influence the neuroinflammation status compared with WT mice after DSS treatment. **(A, B)** Diagrams, representative images (A), and quantitative analysis (B) of Iba1-positive cells in mPFC. **(C, D)** Diagrams, representative images (C), and quantitative analysis (D) of Iba1-positive cells in the amygdala. **(E, F)** Diagrams, representative images (E), and quantitative analysis (F) of Iba1-positive cells in the ventral hippocampus (including CA1, DG, and CA3 areas). **(G, H)** Diagrams, representative images (G), and quantitative analysis (H) of Iba1-positive cells in the dorsal hippocampus (including CA1, DG, and CA3 areas). Scale bars = 200 μm for lower magnification, and 100 μm for the higher magnification. *n* = 4/group. Data are presented as mean ± SEM. Statistical comparisons were performed using two-way ANOVA. *P < 0.05, **P < 0.01. **Figure S8.** mRNA expression levels of hypothalamic neuropeptides and hierarchical clustering of the 934 overlapping genes. **(A)** Hierarchical clustering heatmap of several hypothalamic neuropeptide gene expression profiles (*Crh*, *Sst*, *Npy*, *Agrp*, *Vip*, *Avp*, *Gal*, *Oxt*, and *Trh*) of WT and ERβ^−/−^ mice under homeostasis conditions and treatment with DSS. *n* = 3/group. **(B)** The gene expression profile of the overlapping genes in hypothalamus of WT and ERβ^−/−^ mice under the homeostasis conditions and DSS treatment. **Figure S9.** Fecal microbiota of SiHo WT, SiHo ERβ^−/−^, CoHo WT, and CoHo ERβ^−/−^ mice before DSS treatment. **(A)** UniFrac distances showing microbiota compositional differences among SiHo WT, SiHo ERβ^−^^/^^−^, CoHo WT and CoHo ERβ^−/−^ mice. **(B)** Taxonomic cladogram obtained using LEfSe analysis. **(C)** Relative abundances of substantially changed bacterial taxa at the genus level. **(D)** Relative abundances of substantially changed bacterial taxa at the family level. Data are presented as boxplots. Statistical comparisons were performed using the non-parametric Wilcoxon rank sum test. *n* = 9/group, except for *n* = 8 for the CoHo WT group. *P < 0.05, **P < 0.01, ***P < 0.001. Supplemental materials and methods.

## Data Availability

All data generated or analyzed during this study are included in this published article and its supplementary information files, and deposited at the NCBI Sequence Read Archive (SRA) under the accession number PRJNA632986.

## Abbreviations

AB-PAS: Alcian blue periodic acid schiff
ACTH: Adrenocorticotropic hormone
Akt2: AKT serine/threonine kinase 2
Avp: Arginine vasopressin
Cdkna1a: Cyclin-dependent kinase inhibitor 1a
CNS: Central nervous system
CoHo: Co-housed
Crh: Corticotropin-releasing hormone
Cxcl1: C-X-C motif chemokine ligand 1
DEGs: Different expressed genes
DSS: Dextran sodium sulfate
ELISA: Enzyme-linked immunosorbent assay
ER: Estrogen receptor
ErbB4: Erb-b2 receptor tyrosine kinase 4
Gsk3b: Glycogen synthase kinase 3 beta
KEGG: Kyoto Encyclopedia of Genes and Genomes
HE: Hematoxylin and eosin
HPA: Hypothalamic-pituitary-adrenal
Iba1: Ionized calcium-binding adapter molecule 1
IBD: Inflammatory bowel disease
Ifng: Interferon gamma
Il: Interleukin
OTUs: Operational taxonomic units
mPFC: Medial prefrontal cortex
NHE3: Na+/H+ exchanger 3
Nlrx1: NLR family member x1
Oxt: Oxytocin
PCoA: Principal component analysis
Pik3ca: Phosphatidylinositol-4,5-bisphosphate 3-kinase catalytic subunit alpha
Pik3r1: Phosphoinositide-3-kinase regulatory subunit 1
PVN: Paraventricular nucleus
RNA-seq: RNA sequencing
SiHo: Single-housed
Tnfa: Tumor necrosis factor alpha
WT: Wild type
