# Supplementary material for: Estrogen receptor β deficiency impairs gut microbiota: a possible mechanism of IBD-induced anxiety-like behavior
Source: Microbiome. 2022 Sep 29;10:160. doi: 10.1186/s40168-022-01356-2 (PMC9520828; doi:10.1186/s40168-022-01356-2)
Supplement: Supplementary file 2 — Additional file 1: Table S1. Scoring system for histological changes in the colon. Table S2. The sequences of primers used in this study. Figure S1. ERβ deficiency did not influence the sensorimotor function, memory function, or social interactions in mice following induced experimental colitis. (A) Nest score in the nest building test was performed among the four groups to detect the sensorimotor functions. (B) Spatial memory was assessed by percentage of spontaneous alterations in the Y maze test. (C) Recognition memory was detected by the discrimination index in the novel object recognition test. (D, E) Time spent in each chamber and time spent in sniffing a novel mouse or novel object were used to test the sociability in the social approach period (D). Social recognition was evaluated by the time spent in each chamber and time sniffing familiar mouse or novel mouse in social novelty period (E). Data are presented as mean ± SEM. Statistical comparisons were performed by two-way ANOVA or paired t-test for the three-chamber test. n = 8/group. *P < 0.05, **P < 0.01. Figure S2. Fecal microbiota of WT and ERβ−/− mice under baseline and inflammatory states at the class and order levels. (A) Bar graph of bacterial abundance at the class level. (B) Relative abundances of substantially changed bacterial taxa at the class level. (C) Bar graph of bacterial abundances at the order level. (D) Relative abundances of substantially changed bacterial taxa at the order level. Data are presented as boxplots. Statistical comparisons were performed using the non-parametric Wilcoxon rank sum test. n = 9/group, except for n = 8 in the WT DSS group. *P < 0.05. Figure S3. ERβ deficiency does not influence gut microbiota composition in adult female mice. (A) Community richness calculated by observed OTUs. (B, C) Principal coordinates analysis of microbial unweighted UniFrac compositional differences (B), quantified by UniFrac distance (C) between WT and ERβ−/− female mice. (D) Taxonomic c [file 40168_2022_1356_MOESM1_ESM.docx]

Supplemental figures


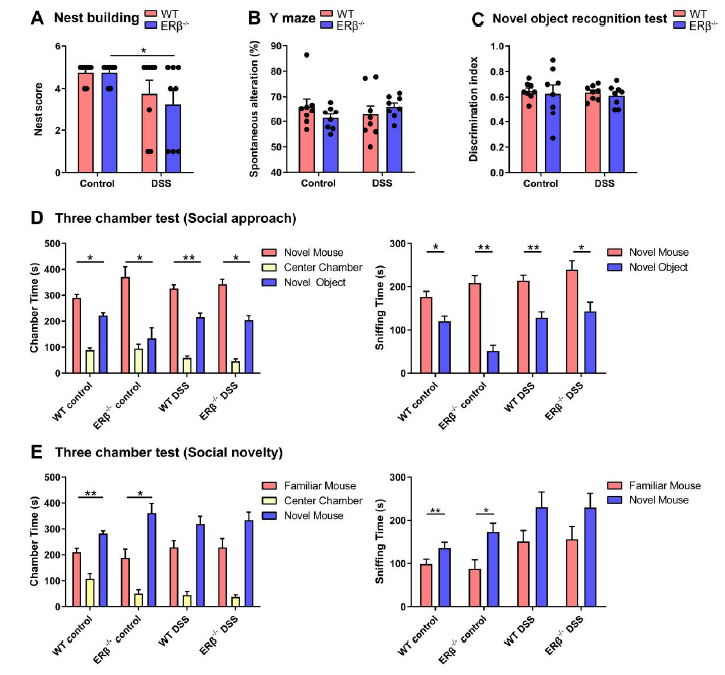


**Figure S1. ERβ deficiency did not influence the sensorimotor function, memory function, or social interactions in mice following induced experimental colitis.**

**(A)** Nest score in the nest building test was performed among the four groups to detect the sensorimotor functions. **(B)** Spatial memory was assessed by percentage of spontaneous alterations in the Y maze test. **(C)** Recognition memory was detected by the discrimination index in the novel object recognition test. **(D, E)** Time spent in each chamber and time spent in sniffing a novel mouse or novel object were used to test the sociability in the social approach period (D). Social recognition was evaluated by the time spent in each chamber and time sniffing familiar mouse or novel mouse in social novelty period (E). Data are presented as mean ± SEM. Statistical comparisons were performed by two-way ANOVA or paired *t*-test for the three chamber test. *n* = 8/group. **P* < 0.05, ***P* < 0.01.


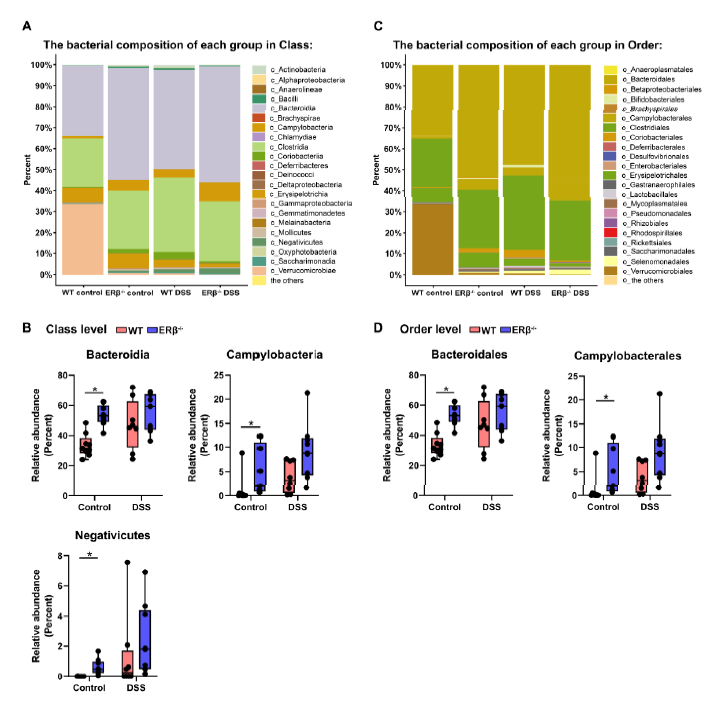


**Figure S2. Fecal microbiota of WT and ERβ^−/−^ mice under baseline and inflammatory states at the class and order levels.**

**(A)** Bar graph of bacterial abundance at the class level. **(B)** Relative abundances of substantially changed bacterial taxa at the class level. **(C)** Bar graph of bacterial abundances at the order level. **(D)** Relative abundances of substantially changed bacterial taxa at the order level. Data are presented as boxplots. Statistical comparisons were performed using the non-parametric Wilcoxon rank sum test. *n* = 9/group, except for *n* = 8 in the WT DSS group. **P* < 0.05.


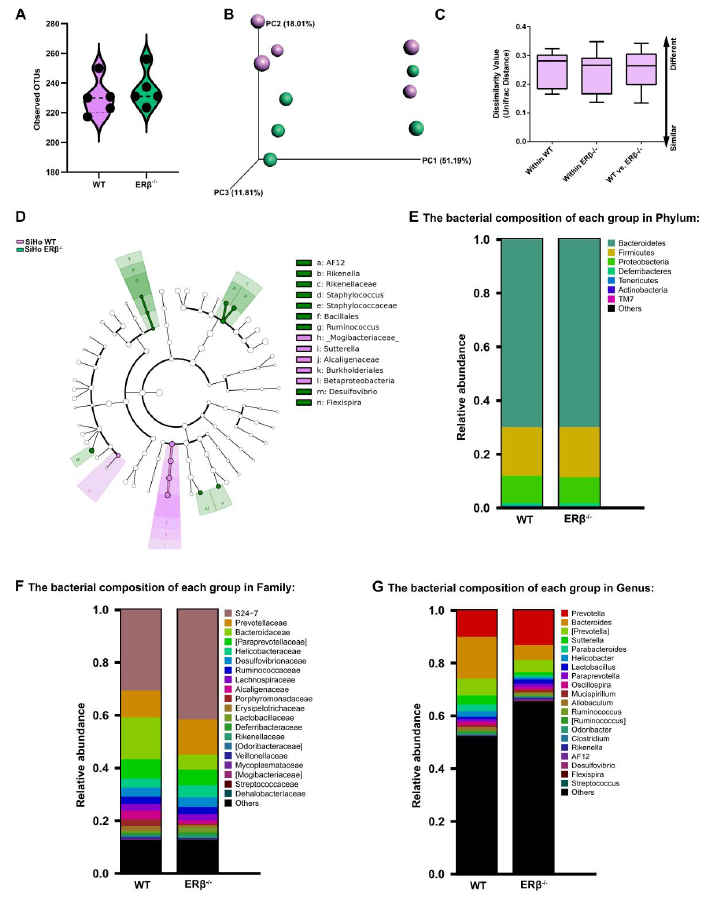


**Figure S3. ERβ deficiency does not influence gut microbiota composition in adult female mice.**

**(A)** Community richness calculated by observed OTUs. **(B, C)** Principal coordinates analysis of microbial unweighted UniFrac compositional differences (B), quantified by UniFrac distance (C) between WT and ERβ^−/−^ female mice. **(D)** Taxonomic cladogram obtained using LEfSe analysis. **(E–G)** Bar graph of bacterial abundances at the phylum (E), family (F), and genus (G) levels. Data are presented as boxplots. Statistical comparisons were performed using the non-parametric Wilcoxon rank sum test. *n* = 5/group.


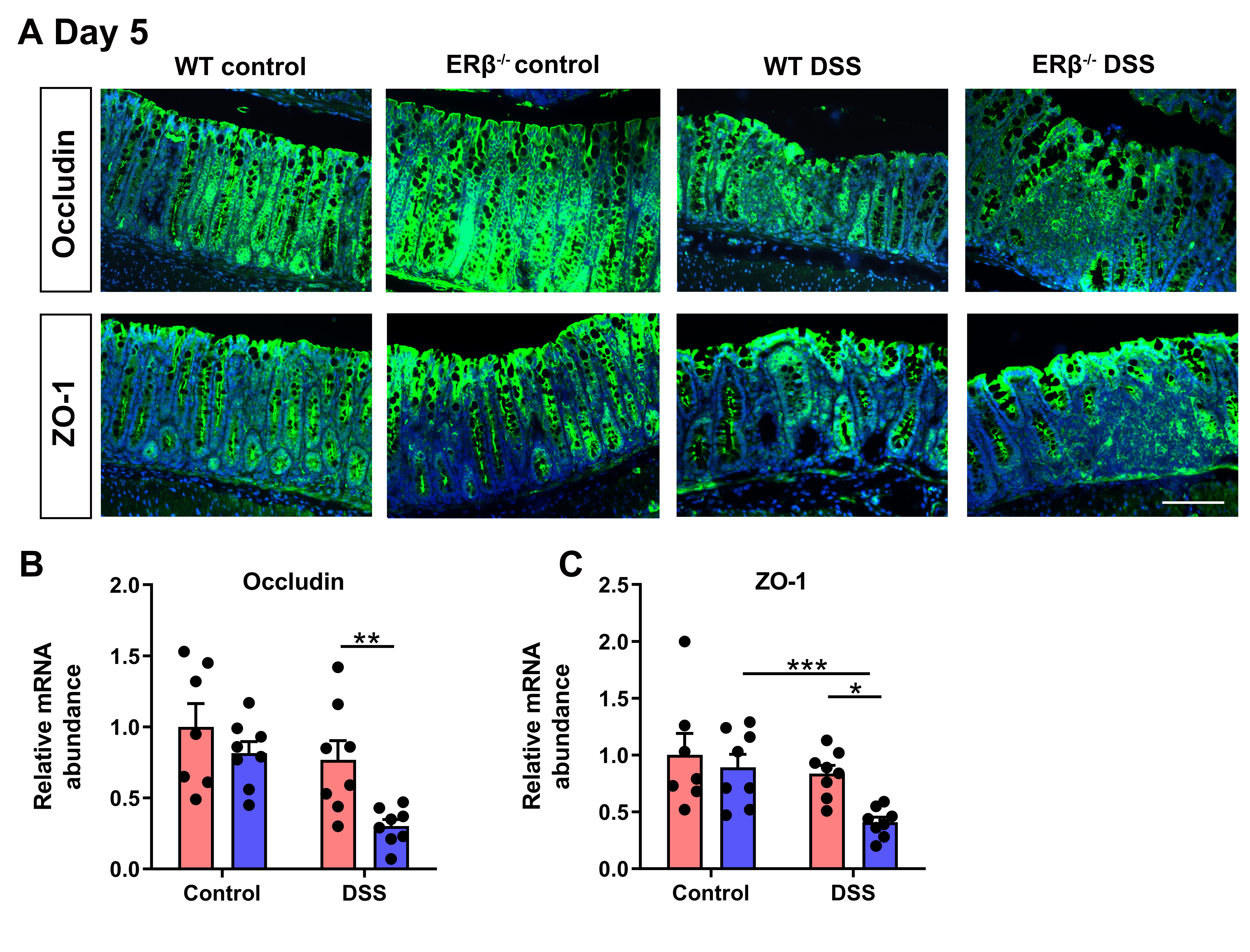


**Figure S4. Tight junctions in WT and ERβ^−/−^ mice under the baseline and inflammatory states on day 5 post-DSS treatment.**

**(A)** Representative images of immunofluorescence staining for tight junction proteins (occludin and ZO-1) in the distal colon of WT and ERβ^−/−^ mice under homeostatic conditions and 5 days following DSS treatment. Scale bar = 100 μm. **(B–C)** Quantitative real-time PCR analysis of mRNA expressions of occludin and ZO-1 in whole colon tissues of WT and ERβ^−/−^ male mice under homeostatic conditions and 5 days following DSS treatment. *n* = 7-8/group. Data are presented as mean ± SEM. **P* < 0.05, ***P* < 0.01.


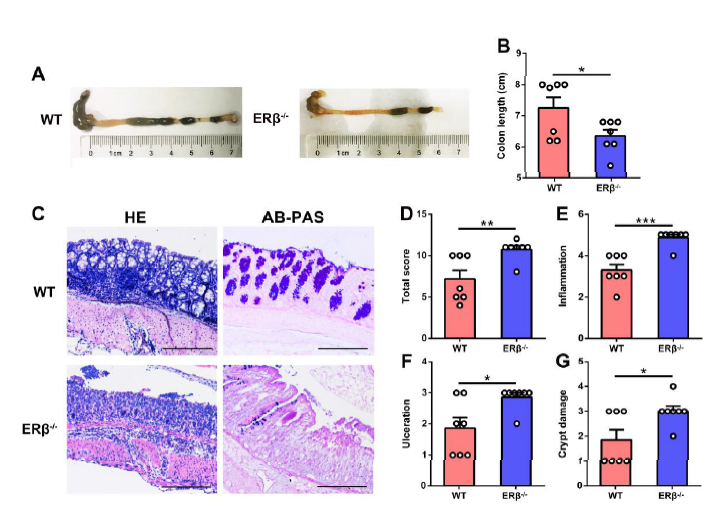


**Figure S5. ERβ deficiency aggravated the development of DSS-induced colitis on day 10 after initial DSS exposure.**

**(A, B)** Mice were sacrificed on day 10 after DSS treatment to measure the colon length. *n* = 7/group. **(C)** Histology of distal colon tissues collected at day 10 was examined by hematoxylin and eosin (HE) and Alcian Blue Periodic Acid Schiff (AB-PAS) staining. Scale bars = 100 µm. **(D–G)** Composite score of histopathology (inflammation, ulceration, and crypt damage scores). *n* = 7/group. **P* < 0.05, ***P* < 0.01, ****P* < 0.001.


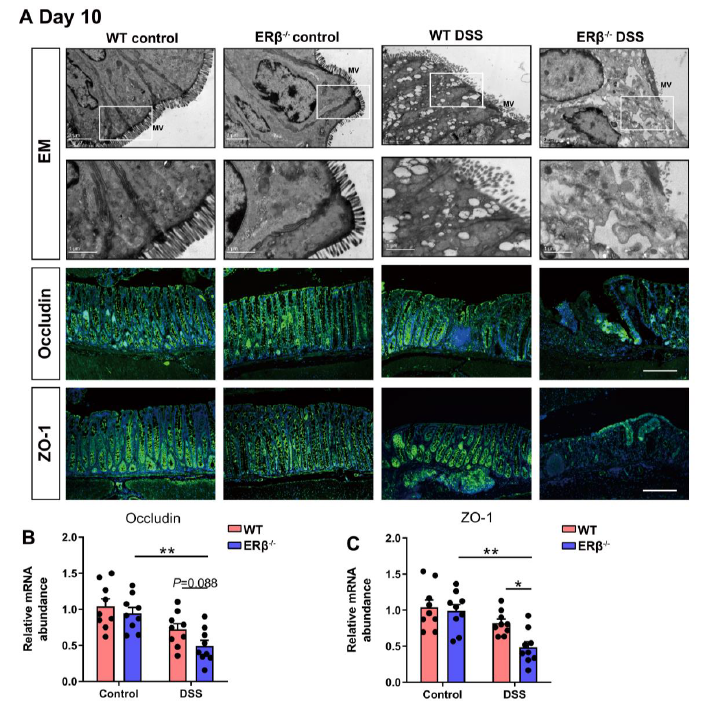


**Figure S6. Tight junctions in WT and ERβ^−/−^ mice under baseline and inflammatory states on day 10 post-DSS treatment.**

**(A)** Tight junctions and villi in the colonic epithelium were examined under an electron microscope (scale bar = 2 or 1 μm as indicated in figure), and representative images of immunofluorescence staining (scale bars = 100 μm) of tight junction proteins (occludin and ZO-1) in the distal colon of WT and ERβ^−/−^ mice under homeostasis conditions and day 10 following DSS treatment. **(B–C)** Quantitative real-time PCR analysis of mRNA expressions of occludin and ZO-1 in whole colon tissues of WT and ERβ^−/−^ male mice under homeostatic conditions and 10 days following DSS treatment. *n* = 9/group. Data are presented as mean ± SEM. **P* < 0.05, ***P* < 0.01.


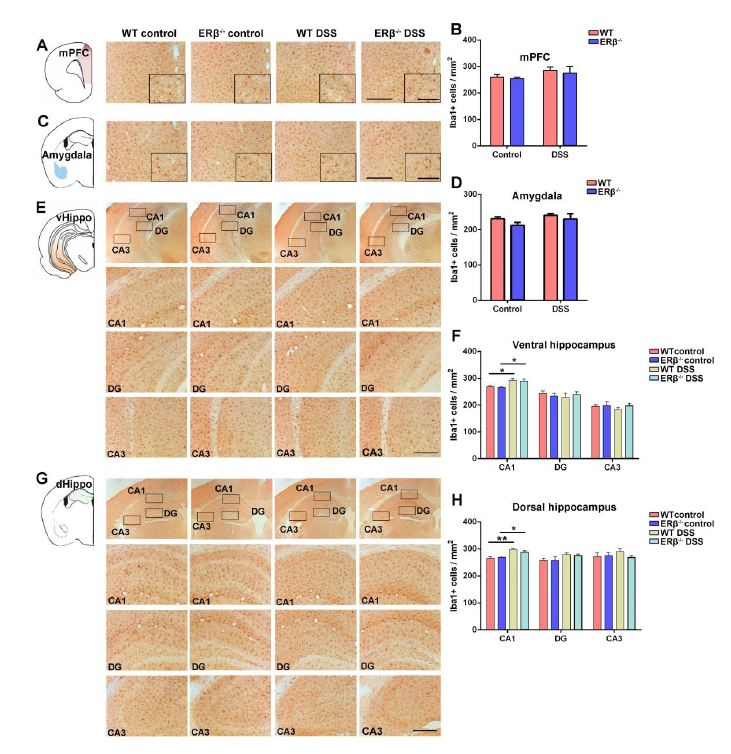


**Figure S7. ERβ deficiency did not significantly influence the neuroinflammation status compared with WT mice after DSS treatment.**

**(A, B)** Diagrams, representative images (A), and quantitative analysis (B) of Iba1-positive cells in mPFC. **(C, D)** Diagrams, representative images (C), and quantitative analysis (D) of Iba1-positive cells in the amygdala. **(E, F)** Diagrams, representative images (E), and quantitative analysis (F) of Iba1-positive cells in the ventral hippocampus (including CA1, DG, and CA3 areas). **(G, H)** Diagrams, representative images (G), and quantitative analysis (H) of Iba1-positive cells in the dorsal hippocampus (including CA1, DG, and CA3 areas). Scale bars = 200 μm for lower magnification , and 100 μm for the higher magnification. *n* = 4/group. Data are presented as mean ± SEM. Statistical comparisons were performed using two-way ANOVA. **P* < 0.05, ***P* < 0.01.


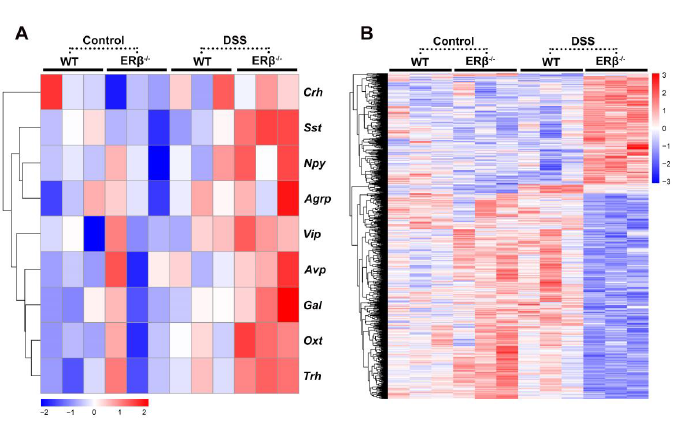


**Figure S8. mRNA expression levels of hypothalamic neuropeptides and hierarchical clustering of the 934 overlapping genes.**

**(A)** Hierarchical clustering heatmap of several hypothalamic neuropeptide gene expression profiles (*Crh*, *Sst*, *Npy*, *Agrp*, *Vip*, *Avp*, *Gal*, *Oxt,* and *Trh*) of WT and ERβ^−/−^ mice under homeostasis conditions and treatment with DSS. *n* = 3/group. **(B)** The gene expression profile of the overlapping genes in hypothalamus of WT and ERβ^−/−^ mice under the homeostasis conditions and DSS treatment.


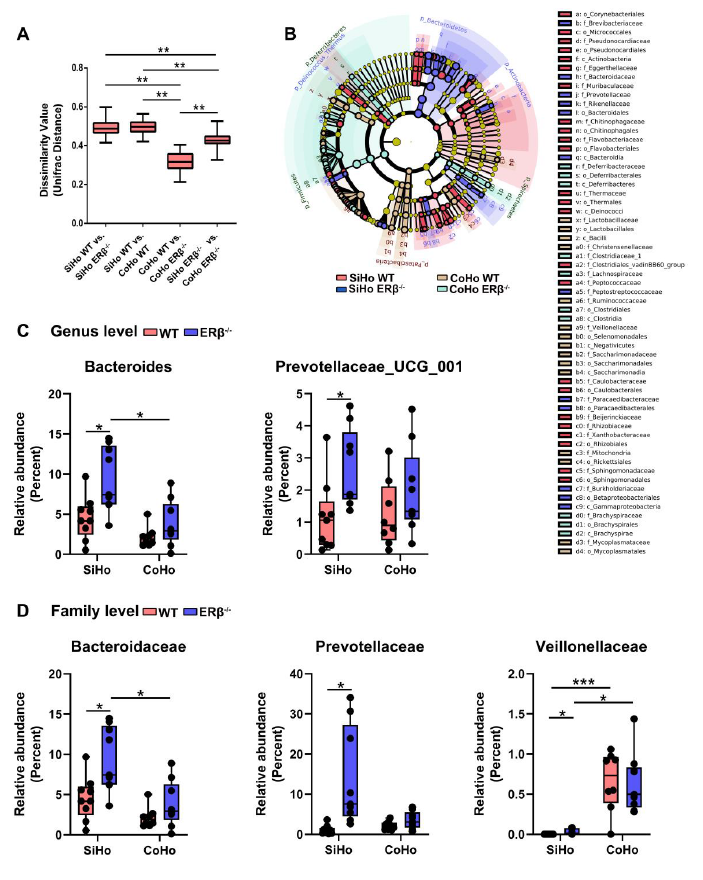


**Figure S9. Fecal microbiota of SiHo WT, SiHo ERβ^−/−^, CoHo WT, and CoHo ERβ^−/−^ mice before DSS treatment.**

**(A)** UniFrac distances showing microbiota compositional differences among SiHo WT, SiHo ERβ^−/−^, CoHo WT and CoHo ERβ^−/−^ mice. **(B)** Taxonomic cladogram obtained using LEfSe analysis. **(C)** Relative abundances of substantially changed bacterial taxa at the genus level. **(D)** Relative abundances of substantially changed bacterial taxa at the family level. Data are presented as boxplots. Statistical comparisons were performed using the non-parametric Wilcoxon rank sum test. *n* = 9/group, except for *n* = 8 for the CoHo WT group. **P* < 0.05, ***P* < 0.01, ****P* < 0.001.

Supplemental materials and methods

**Behavioral assays**

Behavioral tests were performed during day 6 to day 10 following DSS exposure and scheduled in order to avoid carry-over effects from prior testing experience.

*Open field*

The open field test was performed in the apparatus (40 cm × 40 cm × 30 cm) made of grey plexiglas. Initially placed in the center zone, the mouse then got access to exploring the whole arena. Noldus Observer software (Ethovision 11.0) was utilized to analyze the total distance traveled, time spent in the center zone and entries to center during the 10-min period.

*Elevated plus-maze*

Elevated plus-maze apparatus comprised 2 open arms (30 cm × 6 cm × 15 cm), 2 closed arms (30 cm × 6 cm × 15 cm) and a central area. Each mouse was allowed to explore the apparatus freely for 10 min with being placed facing to an open arm initially in the central zone. When 4 paws of the mouse were within the arm, the mouse was seen entirely entering the arm. The time spent in the open arms and number of entries to the open arms were analyzed by Noldus Observer software (Ethovision 11.0).

*Light-dark transitions*

The light-dark box comprised 2 rectangular chambers (light box: length 27 cm, width 27 cm and height 30 cm; dark box: length 18 cm, width 27 cm and height 30 cm) [1]. The mouse was placed in the bright chamber with its back to the opening and allowed to explore the whole box freely for 10 min. The time spent in the dark side and total number of side transitions were recorded using Noldus Observer software (Ethovision 11.0).

*Tail suspension test*

The mice were singly suspended by a tape stuck 1 cm to the tip of the tail and 50 cm height above the ground. The duration of immobility in the last 4 min of the 6-min test was recorded.

*Forced swimming test*

Each mouse was individually placed in a water tank (20 cm height × 14 cm diameter) containing 10 cm of water at 25 °C for 6 min. The floating time, during which the mouse only kept inactive with its head on the surface and slight movements, was recorded to determine the duration of immobility in the last 4 min.

*Nest building*

Each mouse was placed in an individual home cage with a piece of cotton pads (2.5 g/5 cm^2^) (Ancare, Bellmore, NY, USA). Scores were defined according to the amount of torn cotton pads and shape of the nest [2].

*Y maze*

Y maze apparatus comprised 3 arms (40 cm × 9 cm × 16 cm). Each mouse was allowed to explore the apparatus freely for 8 min with being placed in the central zone of Y maze. The spontaneous alteration was calculated as: number of triads containing entries into all 3 arms/maximum possible alternations (the total number of arms entered - 2) × 100% [3].

*Novel object recognition*

The test was performed in the open-field apparatus (40 cm × 40 cm × 30 cm). Firstly, mice were placed in the open and empty field for 30 min in the habituation period. Twenty-four hours after habituation, 2 identical objects were placed in the open field and mice were allowed to explore the objects for 10 min. After a 2 hours interval, 1 novel object was used to replace 1 object that used during habituation period, and mice were placed in the open field to explore the novel or familiar object for 10 min. The discrimination index was calculated as: time exploring novel object/(time exploring novel object + time exploring familiar object) × 100%.

*Three chamber test*

The three chamber test was performed in a rectangular socialization apparatus (60 cm × 40 cm × 22 cm) with 3 chambers as previously described [4]. Firstly, each mouse was place in the middle chamber and allowed to explore the apparatus freely for 10 min during the habituation period. In the social approach period, an unfamiliar C57BL/6 male mouse (novel mouse) was placed in one side of the apparatus, and a novel object was placed in the opposite side. Then, the tested mouse was allowed to explore the apparatus for 10 min. In the social novelty period, another unfamiliar C57BL/6 male mouse (novel mouse) was placed in one side of the apparatus, and the mouse that used in the social approach period (familiar mouse) was placed in the opposite side. Next, each mouse was placed in the apparatus to explore the box for 10 min. The time in each chamber and sniffing time for mouse or object were recorded using Noldus Observer software (Ethovision 11.0).

**16S rRNA gene sequencing and data analysis**

The total microbial genomic DNA were extracted from mouse fecal pellets using DNeasy PowerSoil Kit (Qiagen, Hilden, Germany), and the V3–V4 regions of bacterial 16S rRNA genes were PCR amplified using modified universal primer pairs. After PCR reaction in triplicate, Agencourt AMPure Beads (Beckman Coulter, Brea, CA, USA) was used to purify the PCR amplicons, followed by quantification using the PicoGreen dsDNA Assay Kit (Invitrogen, Carlsbad, CA, USA). Based on the Illumina MiSeq platform, paired-end 2 × 300 bp sequencing was performed using the MiSeq Reagent Kit. Sequence data were processed by open reference out picking, which shared 97% of sequence similarity to the SILVA132 database, and analyzed using QIIME and R packages (v. 3.2.0).

**Histopathology**

Colonic and hypothalamic tissues were fixed in 4% paraformaldehyde (PFA)/PBS overnight and embedded in paraffin. The samples were cut into 5 μm. The degree of colonic injury was coded and assessed in a blinded fashion by hematoxylin and eosin (HE) and alcian-blue periodic acid schiff (AB-PAS) staining colonic sections based on a scale that grades the extent of inflammatory infiltration (0–5), ulceration (0–3) and crypt damage (0–4) [5]. The detailed scoring system for histological changes in the colon were presented in Table S1.

**Quantitative real-time PCR**

The total RNA of colon and hypothalamic tissues was extracted using an Ultrapure RNA kit (CWBIO), which reverse transcribed the RNA concentrations into cDNA following the instructions of the PrimeScript RT Reagent Kit (TaKaRa, Shiga, Japan). The qRT-PCR was performed and analyzed using the CFX96 Real-Time PCR system (Bio-Rad Laboratories, Hercules, CA, USA). The experiments were performed in triplicate and the averaged relative levels of target mRNAs were normalized to the GAPDH expression level. The primer sequences used are listed in Table S2.

**Immunohistochemistry**

Paraffin sections were processed for antigen retrieval, as described previously [4], and incubated with primary antibodies in 1% bovine serum albumin (BSA) overnight at room temperature. Colonic sections were incubated with anti-occludin and anti-ZO-1 (Invitrogen) as well as anti-F4/80 (Biolegend) antibodies, and hypothalamic sections were incubated with anti-corticotropin releasing hormone (Crh) (Sigma-Aldrich, St Louis, MO, USA), anti-arginine vasopressin (Avp) (Santa Cruz Biotechnology, Santa Cruz, CA, USA), anti-oxytocin (Oxt) (Chemicon), and anti-ErbB4 (Invitrogen). Microglia were marked by anti-ionized calcium-binding adapter molecule 1 (Iba1) (Dako) using cryostat sections.

For immunohistochemical staining, sections were incubated with the corresponding secondary antibody (1:200, room temperature), followed by incubation with the avidin-biotin-peroxidase complex and 3,3-diaminobenzidine tetrahydrochloride as the chromogen. For immunofluorescence staining, sections were incubated with 488- or Cy3-conjugated secondary antibodies (1:500, room temperature) and then treated with 4’, 6-diamidino-2-phenylindole (DAPI, Sigma-Aldrich). The images were captured using a Zeiss microscope and an Axiocam HR camera and Axiovision software were used (both Carl Zeiss; 20× magnification).

The paraventricular nucleus (PVN) of hypothalamus was selected for analysis and identified using a standard mouse brain atlas [6]. Crh-, Avp-, Oxt-, and ErbB4-positive cells were manually counted bilaterally in three representative brain sections for each mouse and normalized to the size of the section.

**Enzyme linked immunosorbent assays**

To determine corticosterone and adrenocorticotropic (ACTH) hormone levels, trunk blood was collected in EDTA coated eppendorf tubes following rapidly decapitated under isoflurane anesthesia from mice. Next, after spinned for 10 min at 4 °C, the serum was collected and stored at -80°C until processing. Corticosterone and ACTH levels were determined by Elisa kits (Enzo) following manufacturers’ guidelines.

**RNA sequencing**

RNA was extracted from the hypothalamus using RNeasy Plus Micro kit (Qiagen). The sequencing libraries were generated using NEBNext Ultra RNA Library Prep Kit for Illumina, and were pooled and sequenced on Illumina NovaSeq 6000 platforms using paired-end 150-bp sequencing. Raw reads with low-quality bases (N > 10%) and adaptor contaminants were removed, and the clean reads were aligned to the mouse reference genome (mm10) using TopHat (v. 2.0.12). The gene count expression level was analyzed using HTSeq v0.6.1, and differentially expressed transcripts and genes were analyzed using the DESeq R package (1.18.0). Genes with *P* values < 0.05 were considered as having significantly differential expression. The KOBAS software was used to test the statistical enrichment of differentially expressed genes in the KEGG pathways between the groups.

**Data availability**

Original transcript profiling and 16S rRNA sequencing data were deposited at the NCBI Sequence Read Archive (SRA) under the accession number PRJNA632986.

**References**

1. Babri S, Doosti MH, Salari AA. Strain-dependent effects of prenatal maternal immune activation on anxiety- and depression-like behaviors in offspring. Brain Behav Immun. 2014; 37:164-76.

2. Deacon RM. Assessing nest building in mice. Nat Protoc. 2006; 1(3):1117-1119.

3. Zhong H, Xiao R, Ruan R, Liu H, Li X, Cai Y et al. Neonatal curcumin treatment restores hippocampal neurogenesis and improves autism-related behaviors in a mouse model of autism. Psychopharmacology. 2020; 237(12):3539-52.

4. Cai Y, Tang X, Chen X, Li X, Wang Y, Bao X et al. Liver X receptor beta regulates the development of the dentate gyrus and autistic-like behavior in the mouse. Proc Natl Acad Sci U S A. 2018; 115(12):E2725-33.

5. Xu J, Zhou L, Ji L, Chen F, Fortmann K, Zhang K et al. The REGgamma-proteasome forms a regulatory circuit with IkappaBvarepsilon and NFkappaB in experimental colitis. Nat Commun. 2016; 7:10761.

6. Franklin K, Paxinos G, Keith B. The mouse brain in stereotaxic coordinates. 2008; 3(2):6.

Supplemental tables

**Table S1.** Scoring system for histological changes in the colon.

| **Score** | **Inflammatory infiltration** | **Ulceration** | **Crypt damage** |
| --- | --- | --- | --- |
| 0 | No infiltrate | None | None |
| 1 | Occasional cell limited to lamina propria | Small, focal ulcers | Some crypt damage, spaces between crypts |
| 2 | Significant presence of inflammatory cells in lamina propria, limited to focal areas | Frequent small ulcers | Larger spaces between crypts, loss of goblet cells, some shortening of crypts |
| 3 | Infiltrate present in both submucosa and lamina propria, limited to focal areas | Large areas lacking surface epithelium | Large areas without crypts, surrounded by normal crypts |
| 4 | Large amount of infiltrate in submucosa, lamina propria and surrounding blood vessels, covering large areas of mucosa |  | No crypts |
| 5 | Transmural inflammation |  |  |

**Table S2.** The sequences of primers used in this study.

| **Gene** | **Primer sense (5’-3’)** | **Primer antisense (5’-3’)** |
| --- | --- | --- |
| *Tnfa* | GATCGGTCCCCAAAGGGATG | TGAGGGTCTGGGCCATAGAA |
| *Il1b* | TGCCACCTTTTGACAGTGATG | AAGGTCCACGGGAAAGACAC |
| *Il6* | GGAGCCCACCAAGAACGATAG | GTGAAGTAGGGAAGGCCGTG |
| *Il17a* | TGATCAGGACGCGCAAACAT | GGTCTTCATTGCGGTGGAGAGT |
| *Occludin* | TGAAAGTCCACCTCCTTACAGA | CCGGATAAAAAGAGTACGCTGG |
| *ZO-1* | GCCGCTAAGAGCACAGCAA | GCCCTCCTTTTAACACATCAGA |
| *Cxcl1* | GGTGTCCCCAAGTAACGGAG | TTGTCAGAAGCCAGCGTTCA |
| *Ifng* | CGGCACAGTCATTGAAAGCC | TGTCACCATCCTTTTGCCAGT |
| *Erbb4* | GCTCGCAAGTGGCTATGGTA | TCGCCAGCTTCATTTTTGGC |
| *Pik3ca* | ATGCCCCCACGAATCCTAGT | GAGGGTATTTCCTGGCCTCTC |
| *Pik3r1* | GTGCGGGCCGTATAGGTTTTA | TGCGACAGTTTCCTTGGCTT |
| *Akt2* | GGTTCGAGAGAAGGCCACTG | GGAAGGGGTGCCTGGTATTC |
| *Gsk3b* | CCCTTCTGTTCGGCTACCTT | AAGCCGGAACCAATCAGAGA |
| *Cdkn1a* | GCAGATCCACAGCGATATCCA | GACAACGGCACACTTTGCTC |
| *Gapdh* | TGGGTGTGAACCACGAGAAA | AAAGTTGTCATGGATGACCTTGG |
